# Supplementary material for: Moving to productivity: The benefits of healthy buildings
Source: PLoS One. 2020 Aug 6;15(8):e0236029. doi: 10.1371/journal.pone.0236029 (PMC7410200; doi:10.1371/journal.pone.0236029)
Supplement: S1 Appendix — (PDF) [file pone.0236029.s001.pdf]

A Appendix

Dutch (original)
Maastricht University en de gemeente Venlo nodigen u uit om deel te nemen aan een onderzoek over werkplek beleving en kwaliteit. Dat doen we door middel van een enquete over uw werkplek en het gebouw waarin die zich bevindt.
De enquete gaat over uw huidige werkplek. Over een paar maanden zullen we u opnieuw vragen om de vragenlijst in te vullen, of u nu gaat verhuizen naar het nieuwe stadskantoor of niet. De informatie wordt gebruikt om gebouwen en werkplekken te verbeteren, niet alleen in Venlo, maar ook in de rest van Nederland en daarbuiten. Het invullen van de vragenlijst duurt ongeveer tien minuten. We danken u heel hartelijk voor uw hulp!
English (translated)
Maastricht University and the municipality of Venlo invite you to participate in a study on workplace experience and quality. We do this by means of a survey about your workplace and the building in which it is located.
The survey is about your current workplace. In a few months we will ask you again to complete the questionnaire, whether you are moving to the new city office or not. The information is used to improve buildings and workplaces, not only in Venlo, but also in the rest of the Netherlands and beyond. It takes approximately ten minutes to complete the questionnaire. We thank you very much for your help!

Figure A.1: Measurements Environmental Conditions

a. CO_2 Measurements

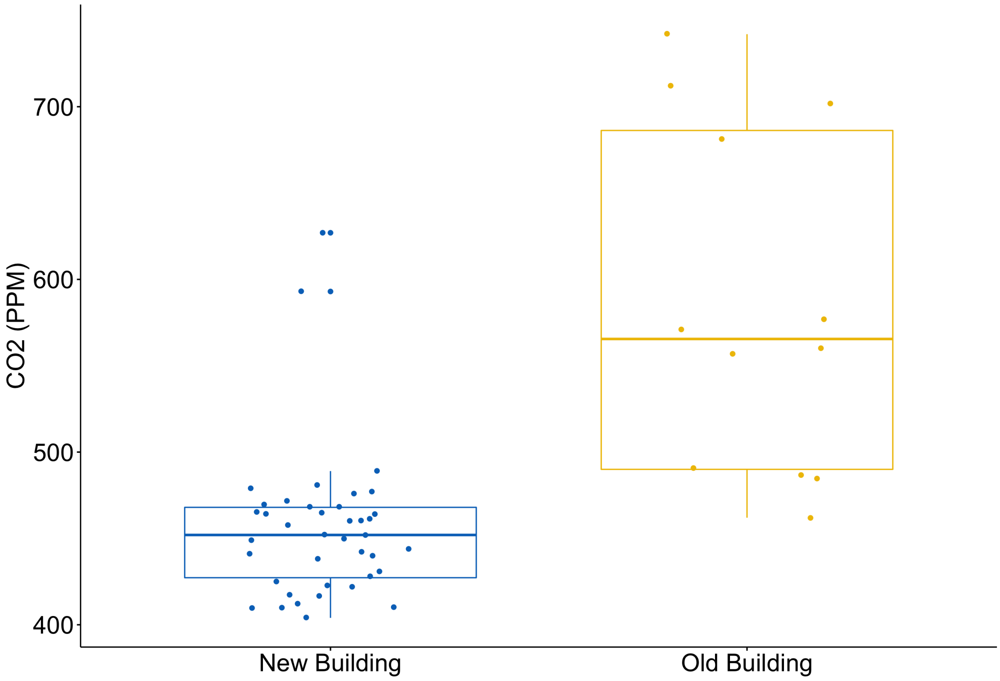

b. Temperature Measurements

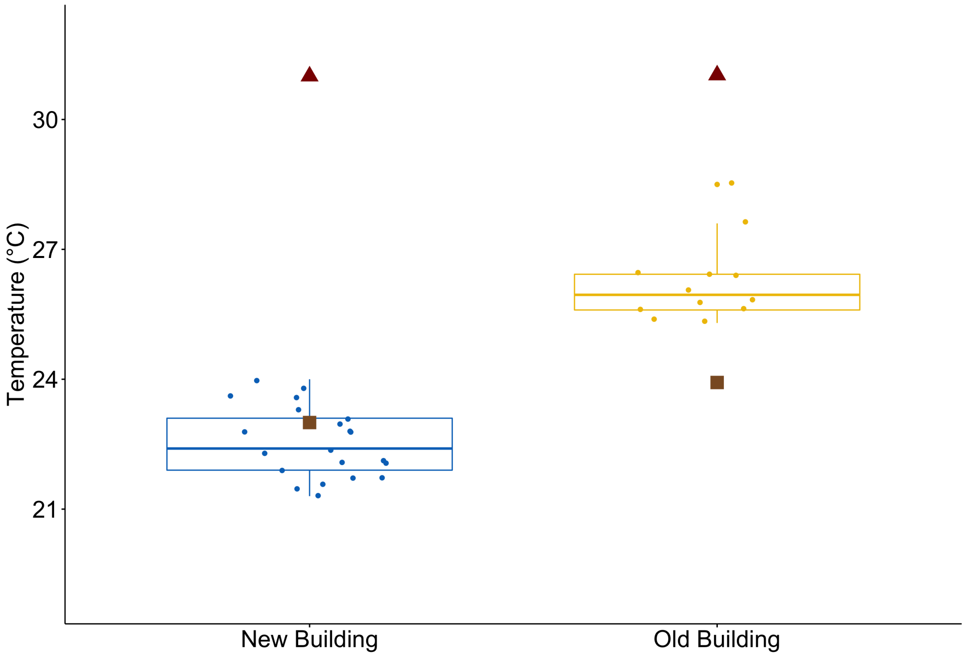

Note: The square (triangle) describes the average (maximum) daily temperature measured by the closest weather station (located in Arcen). Figures based on author's own calculations, based on data retrieved from measurement campaigns undertaken by a professional building surveyor, upon request of the municipality. The measurement campaign in the old building covered four days (before the workers were relocated, from 17/07/2016 to 22/07/2016). In the new building, there is data available from multiple measurement campaigns distributed over all of 2017. For comparison purposes, the distribution of temperatures in the new building is based on a day in 2017 that matches the average peak temperature during the measurement campaign in the old building (21/06/2017). The distribution of CO2 is based on the days that matches the dates in the measurement campaign in the old building to ensure that the occupancy levels are comparable (19/07/2017 to 21/07/2017). Outdoor temperatures are retrieved from The Royal Netherlands Meteorological Institute (KNMI).

Table A.1: Description Questions in Survey

Variable Name	Survey Question
A. Indoor Environmental Condition	
Temperature Quality (Dissatisfaction)	How satisfied are you with the temperature at your workplace?
Temperature Quality (Hinders Work)	In general: does the thermal comfort at your workplace support or hinder your work?
Air Quality (Dissatisfaction)Â	How satisfied are you with the air quality at your workplace (i.e. stuffy/stale air, air cleanliness, odors)?
Air Quality (Hinders Work)	In general: does the air quality at your workplace support or hinder your work?
Light Quality (Dissatisfaction)	How satisfied are you with the amount of light in your workplace?
Views (Dissatisfaction)	How satisfied are you with the visual comfort at your workplace (glare, reflections, contrast ratios)?
Light Quality (Hinders Work)	In general: does the light in your workplace support or hinder your work?
Noise (Dissatisfaction)	How satisfied are you with the noise level at your workplace?
Privacy (Dissatisfaction)	How satisfied are you with the acoustic privacy at your workplace (possibility of having conversations without colleagues listening in)?
Noise (Hinders Work)	In general: does the noise at your workplace support or hinder your work?
B. Office Layout	
Space Available (Dissatisfaction)	How satisfied are you with the space available for carrying out your work and storing work equipment?
Visual Privacy (Dissatisfaction)	How satisfied are you with the visual privacy at your workplace?
Interaction Colleagues (Dissatisfaction)	How satisfied are you with the opportunities for interaction with your colleagues?
Office Layout (Hinders Work)	In general: does the workplace structure support or hinder your work?
Furniture Comfort(Dissatisfaction)	How satisfied are you with the comfort of your office furniture and equipment (eg chair, desk, computer)?
Adaptable Furniture (Dissatisfaction)	How satisfied are you with the options to adjust your furniture to your needs?
Color Furniture (Dissatisfaction)	How satisfied are you with the color and quality of floor coverings, furniture and other surface materials?
Furniture And Equipment (Hinders Work)	In general: Does the office furniture and equipment support or hinder your work?

Note: All questions are based on 7-point Likert scales, from satisfied (1) to dissatisfied (7).

Table A.2: Description Job Satisfaction Scales in Survey

	Non-Relocated (N=247)	Relocated (N=326)	Diff.	Loadings PCA
At work I am full of energy	2.14	2.05	0.09	-0.299
When I work I feel fit and strong	2.10	2.00	0.10	-0.295
I am enthusiastic about my job	1.78	1.73	0.05	-0.311
My work inspires me	1.90	1.86	0.04	-0.316
When I get up in the morning, I feel like going to work	2.00	1.94	0.05	-0.281
When I am working very intensively, I feel happy	1.85	1.85	-0.01	-0.301
I am proud of the work I do	1.78	1.75	0.02	-0.300
I am completely absorbed in my work	2.06	2.00	0.06	-0.308
I can work for a long time	2.17	2.23	-0.06	-0.263
My work thrills me	2.91	3.19	-0.28	-0.259
At work I have a great mental resilience	2.04	2.15	-0.10	
I always persevere at work, even when things go wrong	1.84	2.01	-0.18	-0.284
Job Satisfaction Index (based on PCA)	-0.26	-0.17	-0.09	

Note: The original question asked respondents *'How satisfied are you with the following aspects of your work on a scale from 1 (daily) to 7 (never)'*. The principal component is calculated as a linear combination of job satisfaction variables, where the weights in that linear combination are equal to the loadings reported in this table.
