# Supplementary material for: Moving to productivity: The benefits of healthy buildings
Source: PLoS One. 2020 Aug 6;15(8):e0236029. doi: 10.1371/journal.pone.0236029 (PMC7410200; doi:10.1371/journal.pone.0236029)
Supplement: S1 File — (ZIP) [file pone.0236029.s002.zip › 03_graphs/ventilatoin.htm]

  


New Municipal Office Venlo, NL


MENU


- Markets
- Services
- Insights
- Projects
- Careers
- Search
- Country
  Global

Close menu

 

- About us
  more

  - Company profile
  - Leadership
  - Corporate governance
  - Innovation
  - Corporate responsibility
  - Integrity
- Newsroom
- Offices
- Contact
- Visit our blog

Close menu

### Choose a country

- Global
- The Netherlands
- Australia
- Belgium
  Language

  - FrançaisNederlands
- Czech Republic
- India
- Indonesia
- Latin America
  Language

  - EnglishSpanish
- Myanmar
- Poland
- South Africa
- Thailand
- Turkey
- United Kingdom
- Viet Nam


# New Municipal Office Venlo, Netherlands

## The Challenge

A new town hall arises in Venlo, on the river Maas. It is in many ways a challenging project. The ambition of the Gemeente Venlo is to apply the principles of Cradle to Cradle (C2C ®) to the design of the building. Important features are an excellent indoor climate and very low energy consumption. Furthermore, the building should be ready for energy- and material neutrality in the near future.  

## Our Solution

The new town hall is part of the planning area De Maaswaard. In this area thermal energy storage (TES) is developed that will also supply the town hall. In addition, we applied the following C2C ® solutions:  

- Ventilation based on the principles of natural circulation. Air will be supplied to a glass house on the roof that purifies the air, the exhaust air disappears through voids and a solar chimney on the roof (see graphic)
- Air quality that is further enhanced  by a green façade
- A helophyte area that purifies the grey water.

  
The building is not only efficient but also effective in using space. A three layered parking garage is planned below the town hall that will make maximum use of space, natural ventilation and daylight.  

## The Outcome

Kraaijvanger Architects designed the building. It is now under construction and will be completed in 2015. An important feature of the building is its adaptability to future demands. The building is now equipped with state of the art facilities. However, part of the design process included the development of scenarios in the fields of energy, air, water, management and materials, in order to meet the goals of energy and material neutrality in the near future.

## Contact us

Nick Hendrikx

Consultant Building Services

Nijmegen, NL

- +31 88 3486 945
- +31 6 15830394
- nick.hendrix@rhdhv.com
- LinkedIn

Send message

Send message

## Related markets

- Buildings

## Related services

- Building Services
- Building Technology
- Energy Efficiency in Container Ports
- nearly Zero Energy Buildings

Back to top

- © Royal HaskoningDHV 2019
- Feedback & Disclaimer
- Privacy Statement
- Contact
