# Supplementary figures and images for: Moving to productivity: The benefits of healthy buildings
Source: PLoS One. 2020 Aug 6;15(8):e0236029. doi: 10.1371/journal.pone.0236029 (PMC7410200; doi:10.1371/journal.pone.0236029)

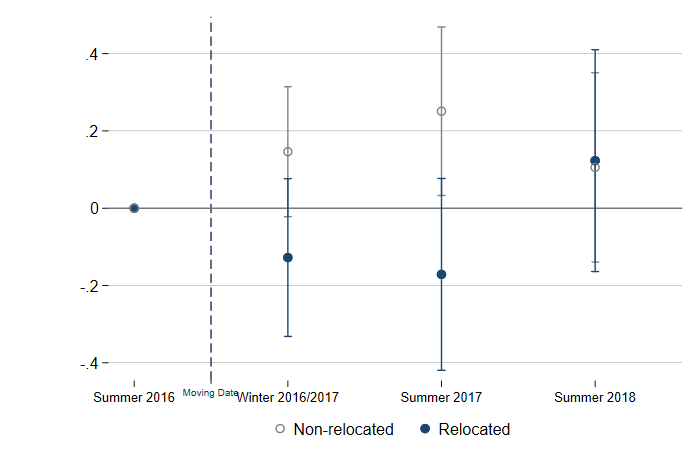

Supplement: S1 File — (ZIP) [file pone.0236029.s002.zip › 03_graphs/dynamic_Q47_7.png]

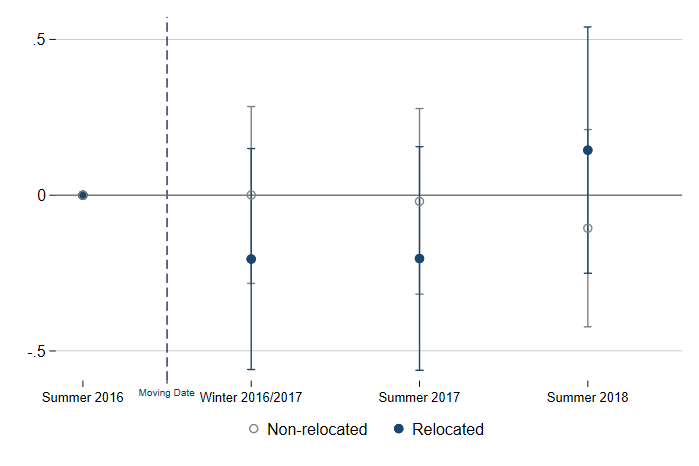

Supplement: S1 File — (ZIP) [file pone.0236029.s002.zip › 03_graphs/dynamic_layout_sc_4.png]

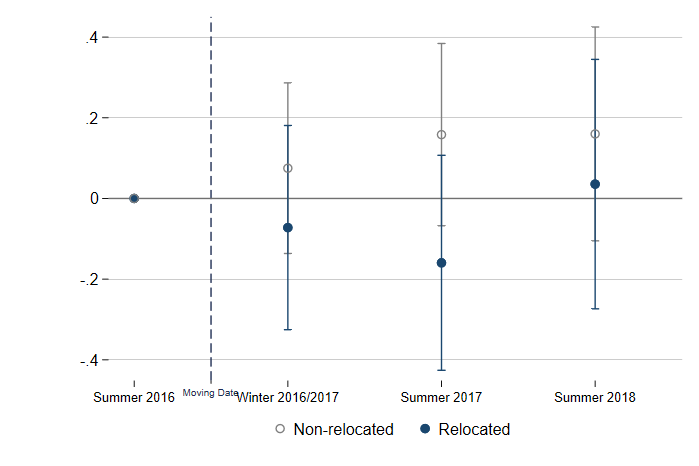

Supplement: S1 File — (ZIP) [file pone.0236029.s002.zip › 03_graphs/dynamic_Q47_11.png]

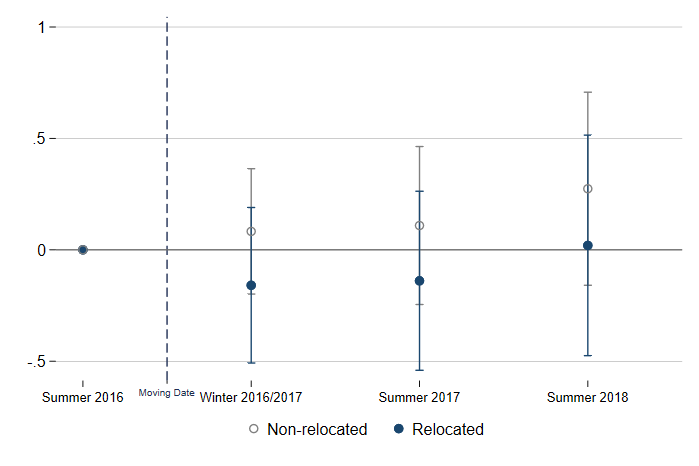

Supplement: S1 File — (ZIP) [file pone.0236029.s002.zip › 03_graphs/dynamic_Q47_10.png]

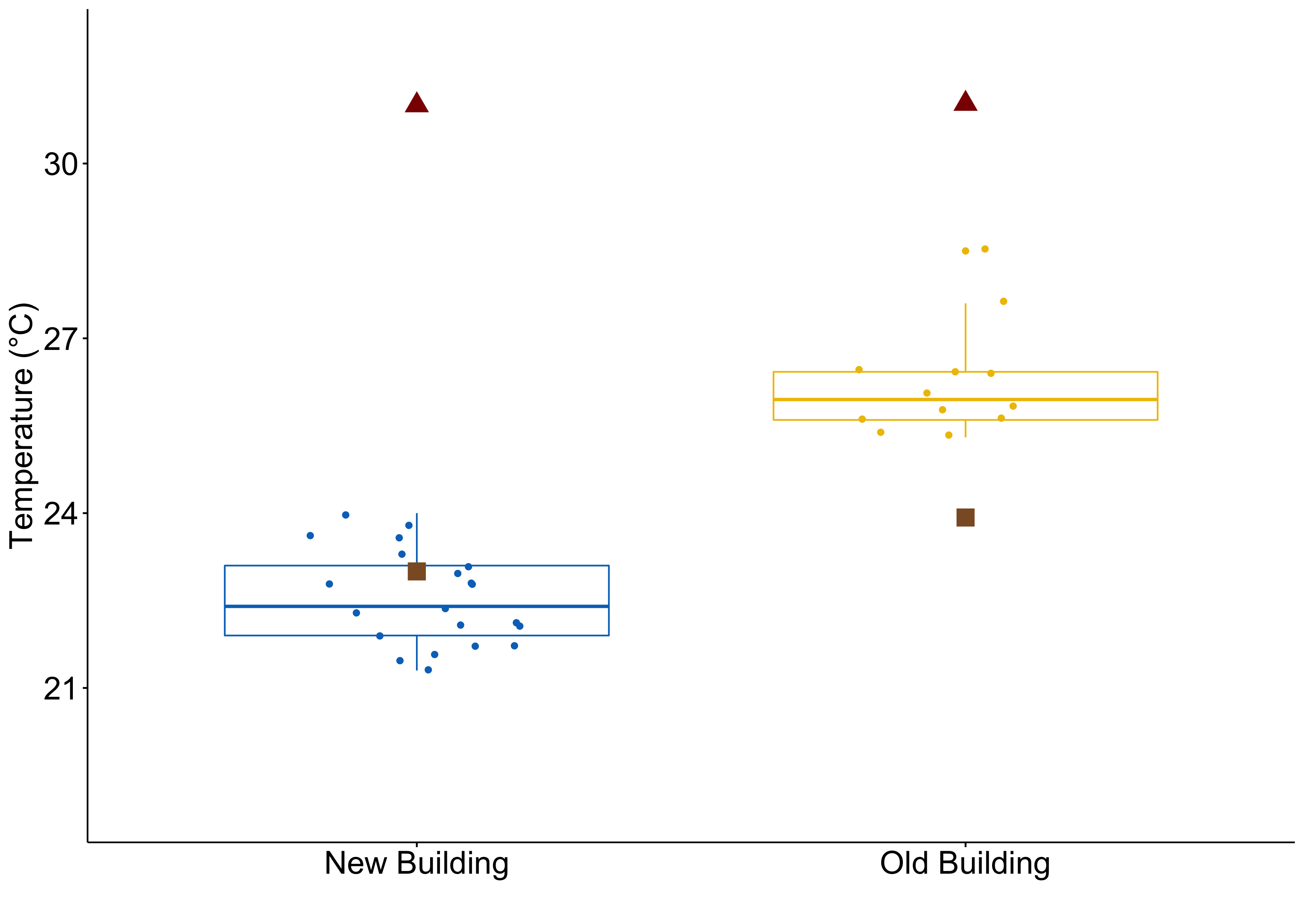

Supplement: S1 File — (ZIP) [file pone.0236029.s002.zip › 03_graphs/meas_temp.png]

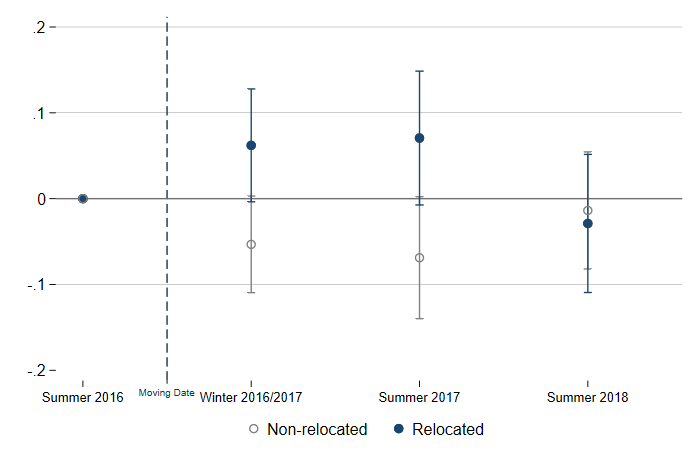

Supplement: S1 File — (ZIP) [file pone.0236029.s002.zip › 03_graphs/dynamic_Q47_7_dm.png]

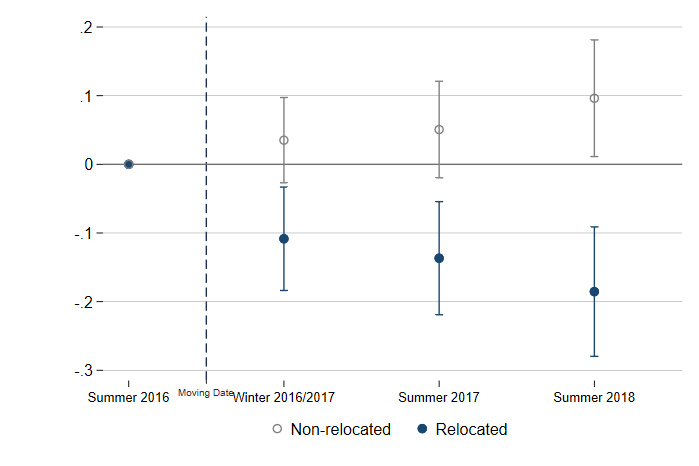

Supplement: S1 File — (ZIP) [file pone.0236029.s002.zip › 03_graphs/dynamic_light_dm_1.png]

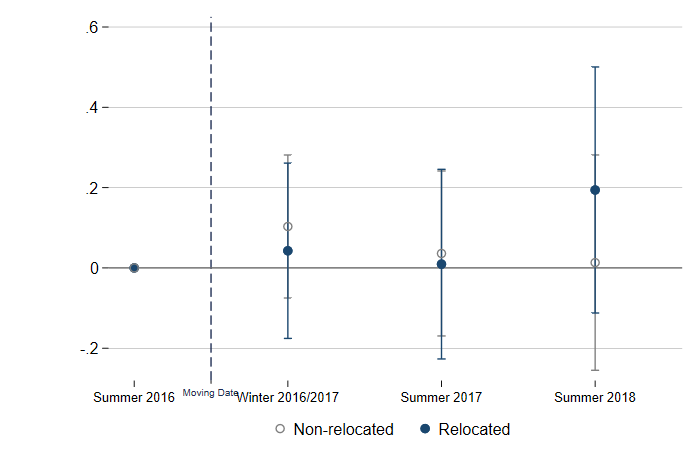

Supplement: S1 File — (ZIP) [file pone.0236029.s002.zip › 03_graphs/dynamic_Q47_6.png]

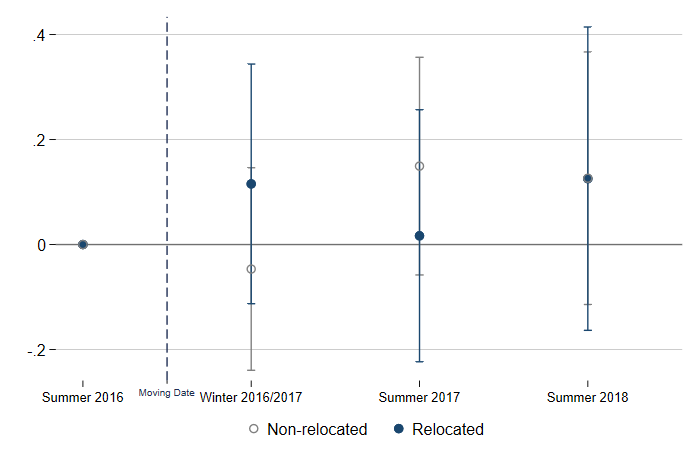

Supplement: S1 File — (ZIP) [file pone.0236029.s002.zip › 03_graphs/dynamic_Q47_4.png]

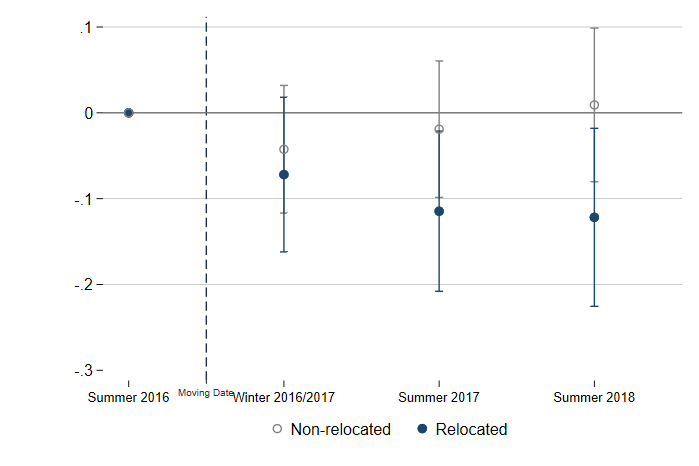

Supplement: S1 File — (ZIP) [file pone.0236029.s002.zip › 03_graphs/dynamic_light_dm_3.png]

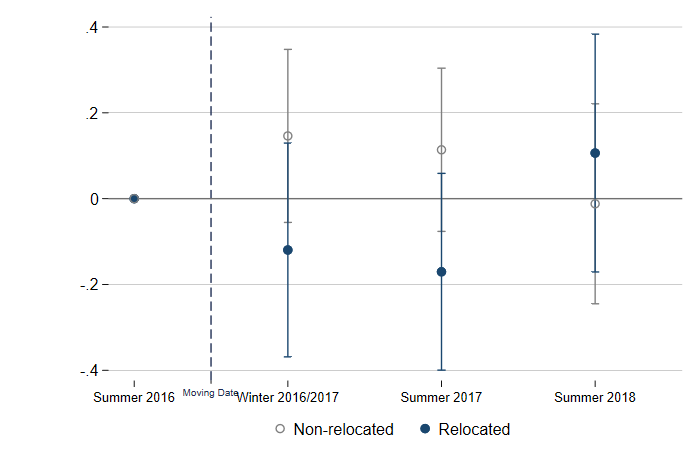

Supplement: S1 File — (ZIP) [file pone.0236029.s002.zip › 03_graphs/dynamic_Q47_12.png]

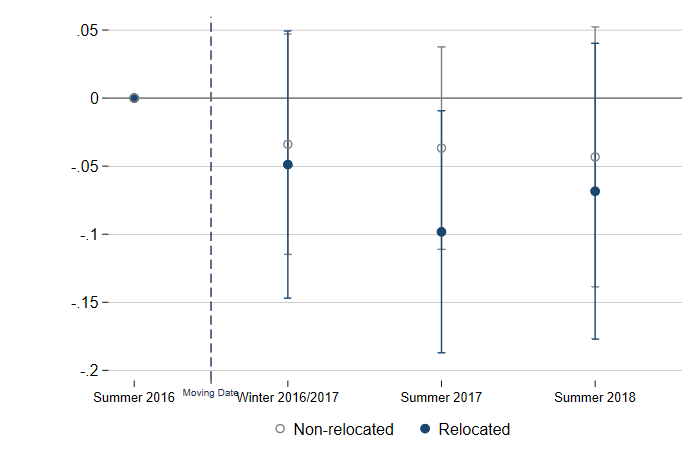

Supplement: S1 File — (ZIP) [file pone.0236029.s002.zip › 03_graphs/dynamic_light_dm_2.png]

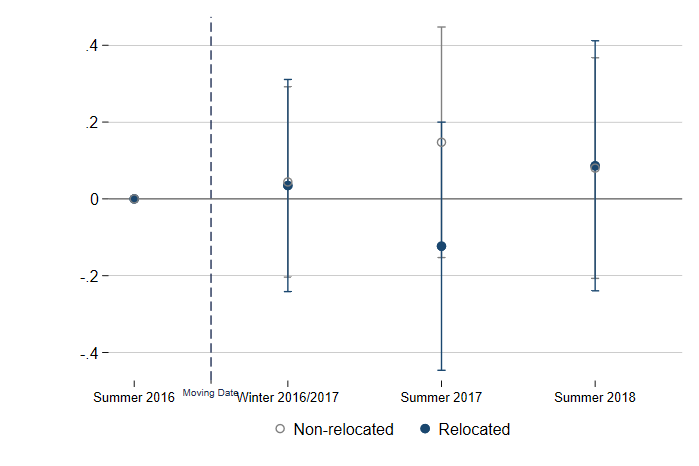

Supplement: S1 File — (ZIP) [file pone.0236029.s002.zip › 03_graphs/dynamic_Q47_5.png]

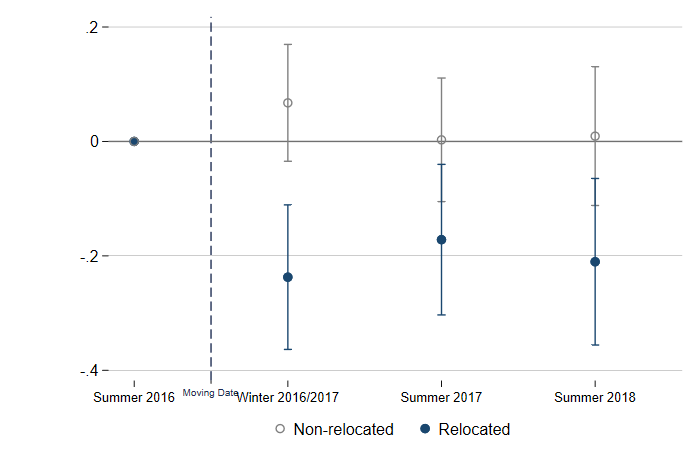

Supplement: S1 File — (ZIP) [file pone.0236029.s002.zip › 03_graphs/dynamic_temp_dm_1.png]

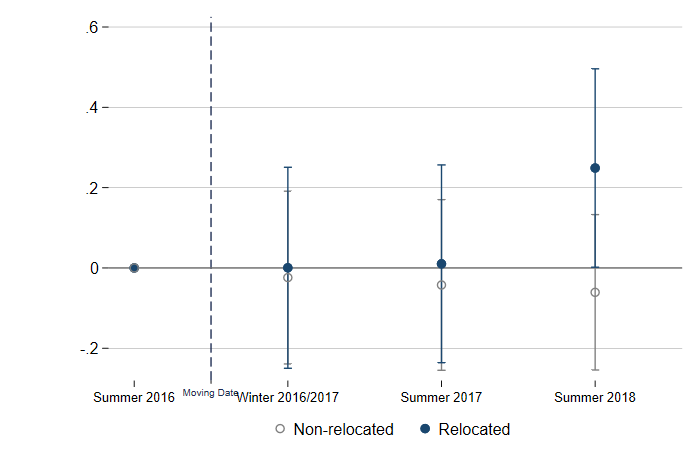

Supplement: S1 File — (ZIP) [file pone.0236029.s002.zip › 03_graphs/dynamic_Q47_1.png]

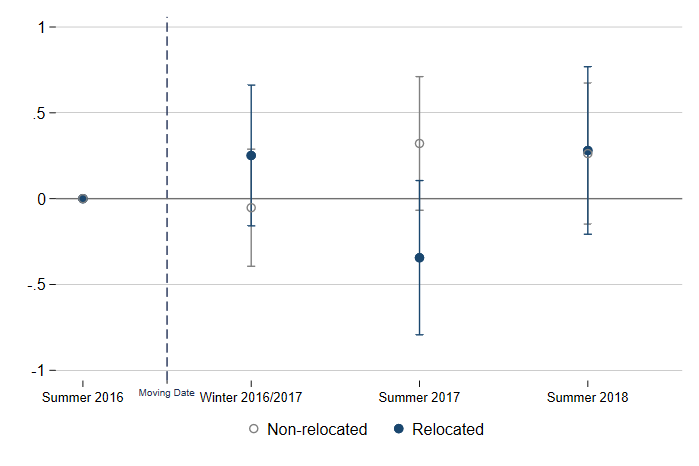

Supplement: S1 File — (ZIP) [file pone.0236029.s002.zip › 03_graphs/dynamic_layout_sc_2.png]

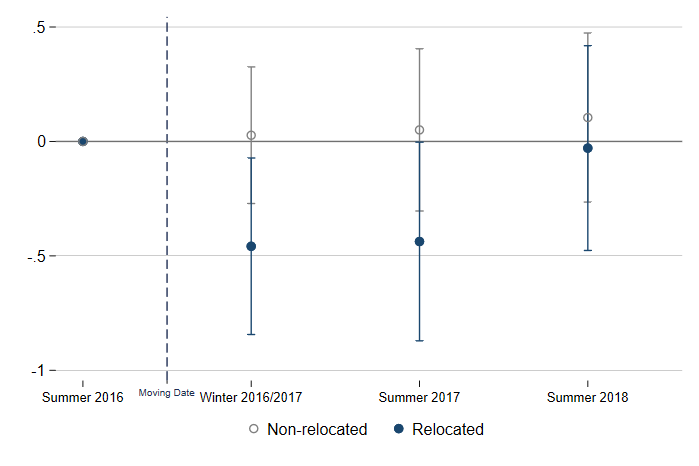

Supplement: S1 File — (ZIP) [file pone.0236029.s002.zip › 03_graphs/dynamic_noise_sc_2.png]

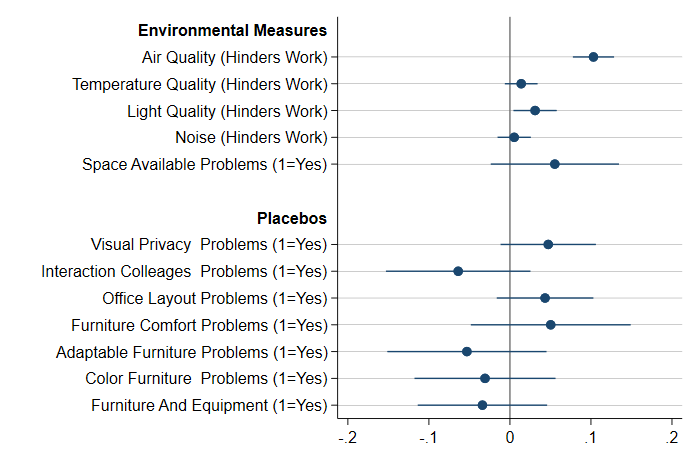

Supplement: S1 File — (ZIP) [file pone.0236029.s002.zip › 03_graphs/sbs_effect_sc_2.png]

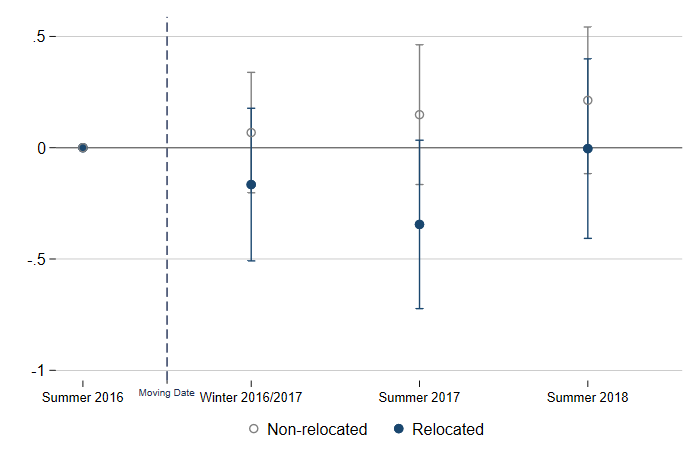

Supplement: S1 File — (ZIP) [file pone.0236029.s002.zip › 03_graphs/dynamic_noise_sc_3.png]

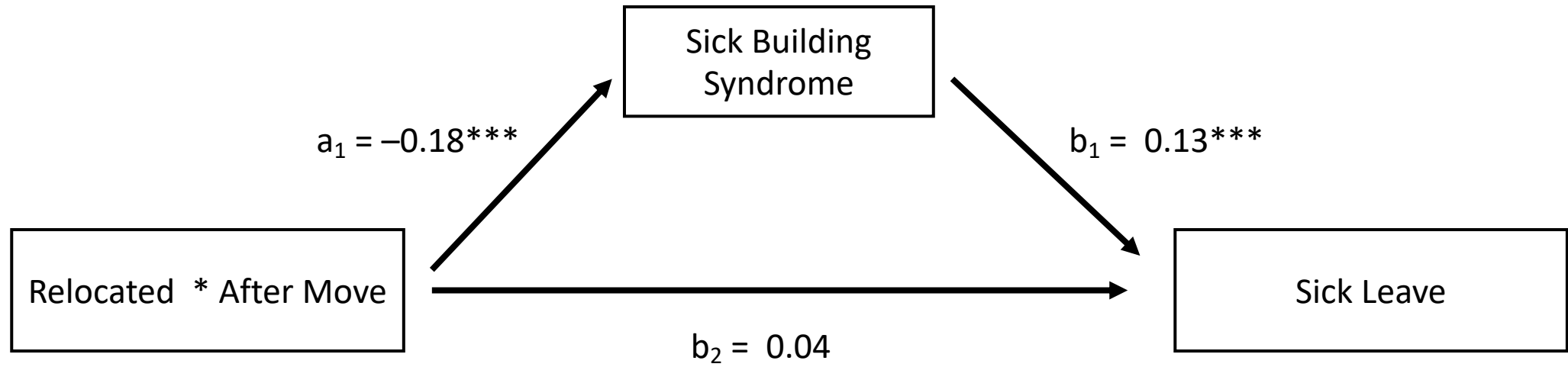

Supplement: S1 File — (ZIP) [file pone.0236029.s002.zip › 03_graphs/mediation_estimates.pdf]

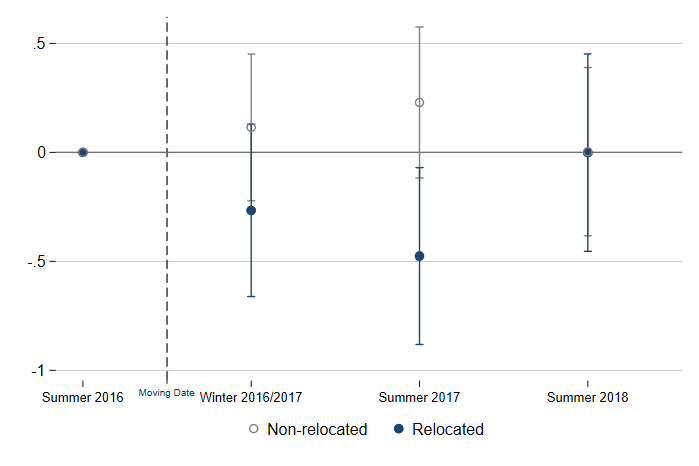

Supplement: S1 File — (ZIP) [file pone.0236029.s002.zip › 03_graphs/dynamic_layout_sc_3.png]

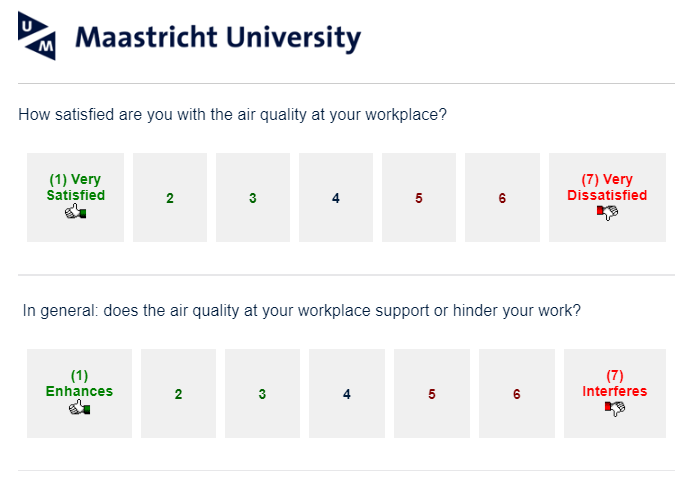

Supplement: S1 File — (ZIP) [file pone.0236029.s002.zip › 03_graphs/air_quality_questions.png]

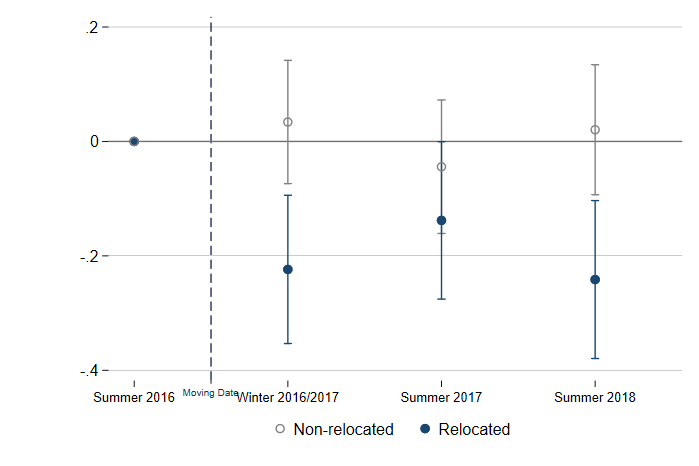

Supplement: S1 File — (ZIP) [file pone.0236029.s002.zip › 03_graphs/dynamic_temp_dm_2.png]

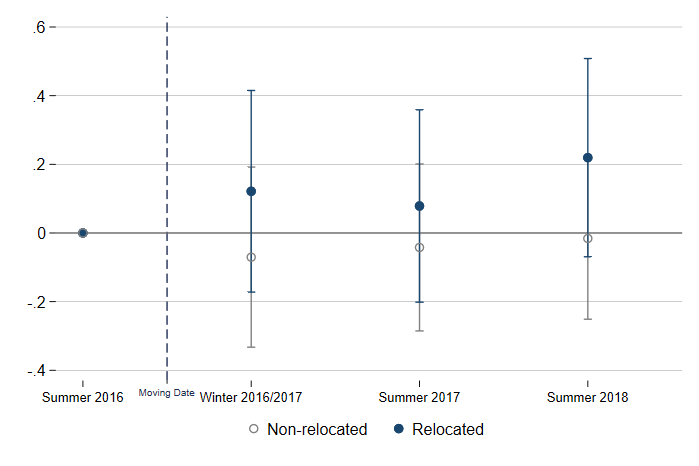

Supplement: S1 File — (ZIP) [file pone.0236029.s002.zip › 03_graphs/dynamic_Q47_2.png]

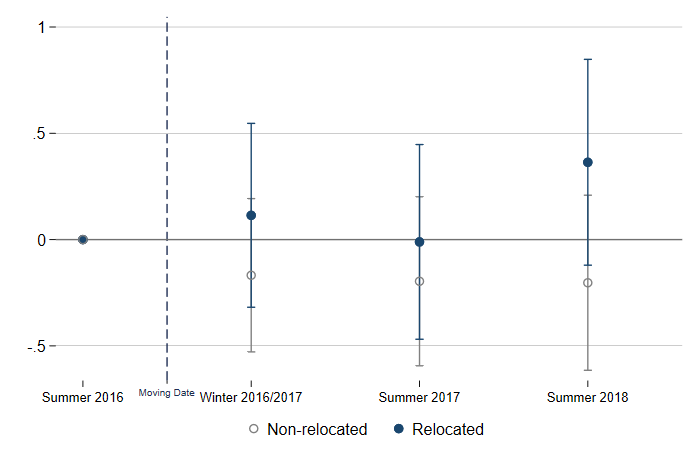

Supplement: S1 File — (ZIP) [file pone.0236029.s002.zip › 03_graphs/dynamic_layout_sc_1.png]

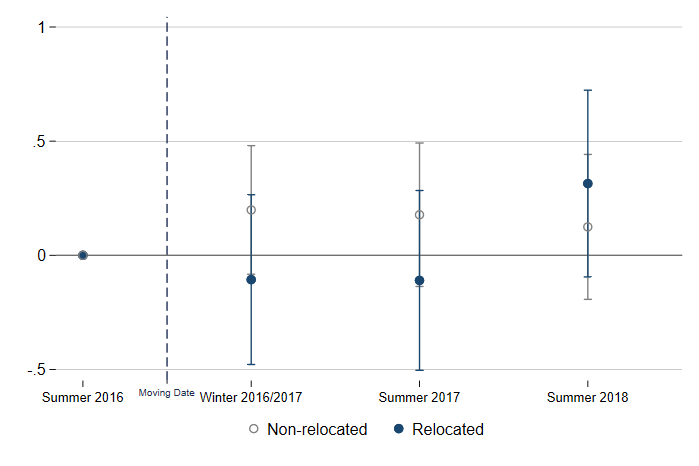

Supplement: S1 File — (ZIP) [file pone.0236029.s002.zip › 03_graphs/dynamic_noise_sc_1.png]

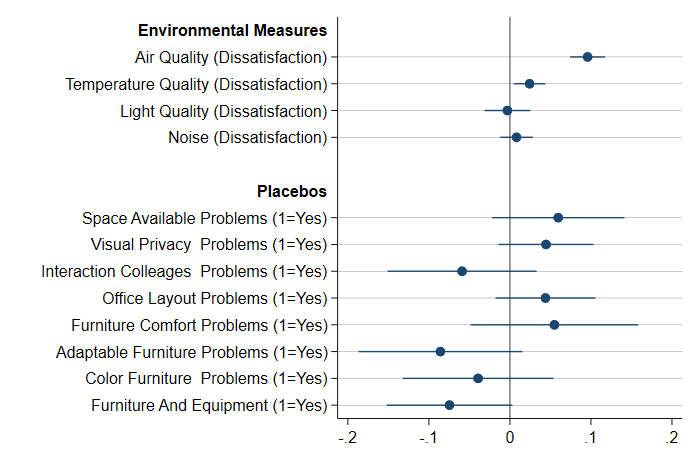

Supplement: S1 File — (ZIP) [file pone.0236029.s002.zip › 03_graphs/sbs_effect_sc_1.png]

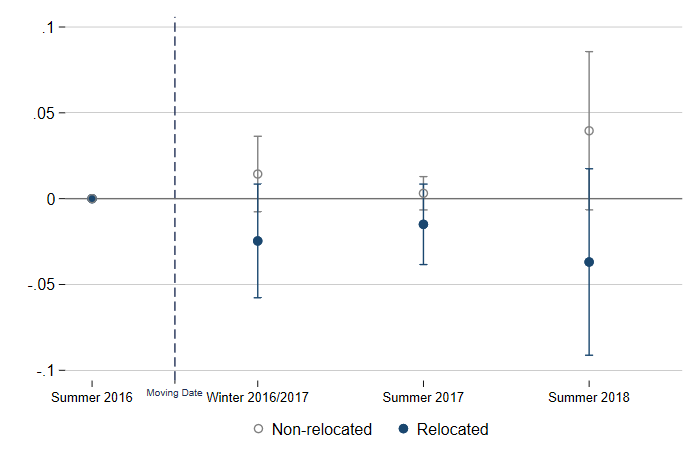

Supplement: S1 File — (ZIP) [file pone.0236029.s002.zip › 03_graphs/dynamic_Q49_2_dm.png]

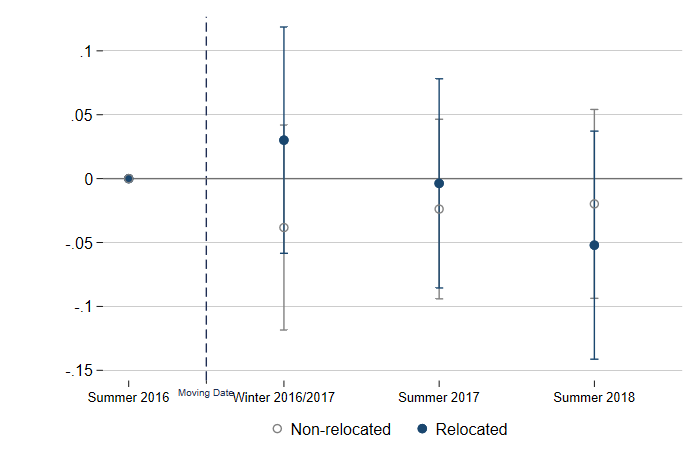

Supplement: S1 File — (ZIP) [file pone.0236029.s002.zip › 03_graphs/dynamic_Q47_9_dm.png]

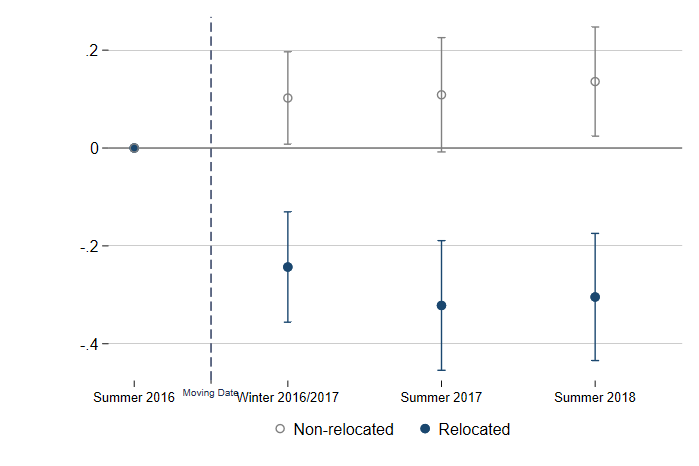

Supplement: S1 File — (ZIP) [file pone.0236029.s002.zip › 03_graphs/dynamic_sbs.png]

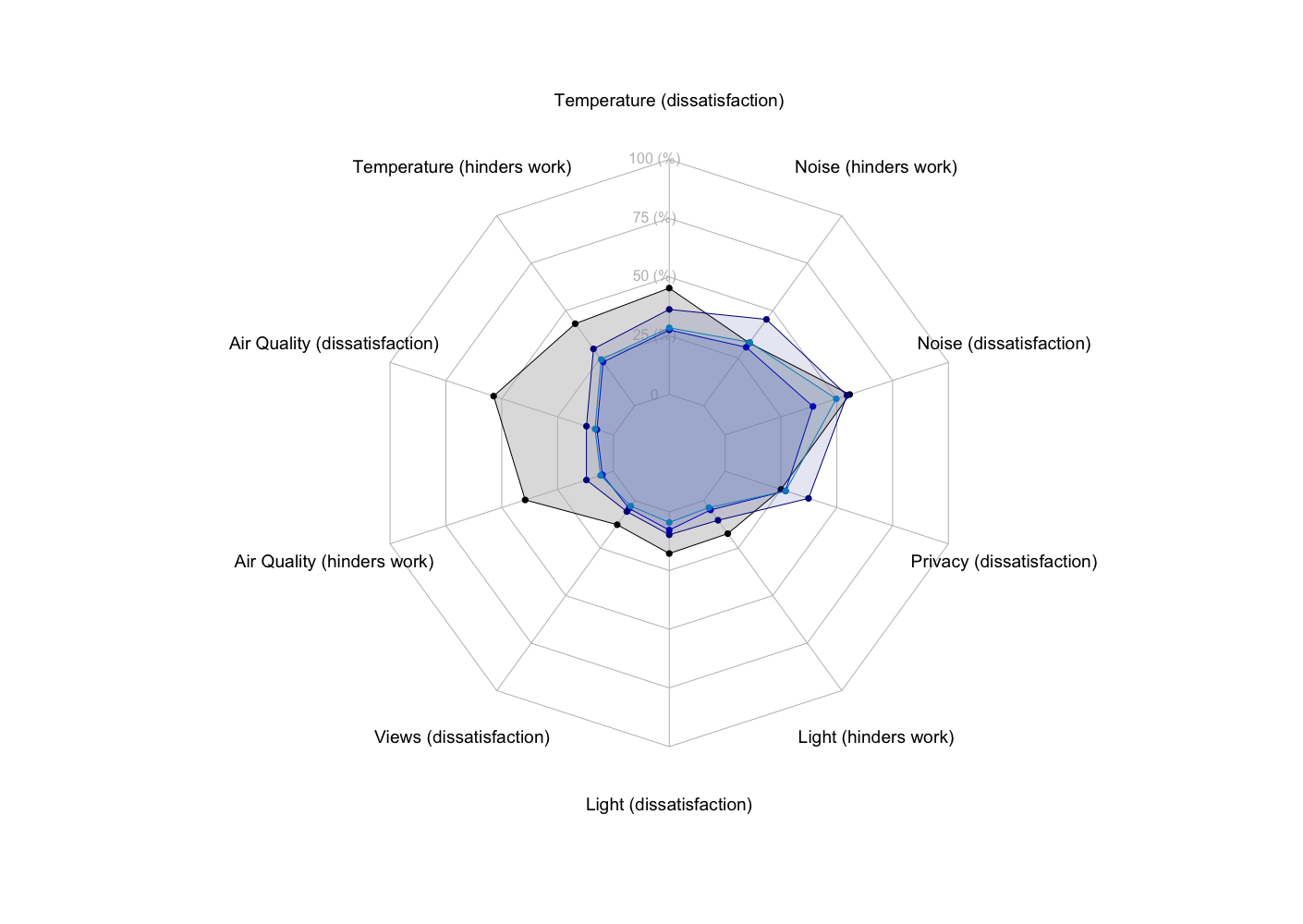

Supplement: S1 File — (ZIP) [file pone.0236029.s002.zip › 03_graphs/radar_treat.png]

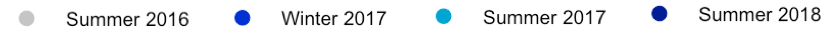

Supplement: S1 File — (ZIP) [file pone.0236029.s002.zip › 03_graphs/legend.png]

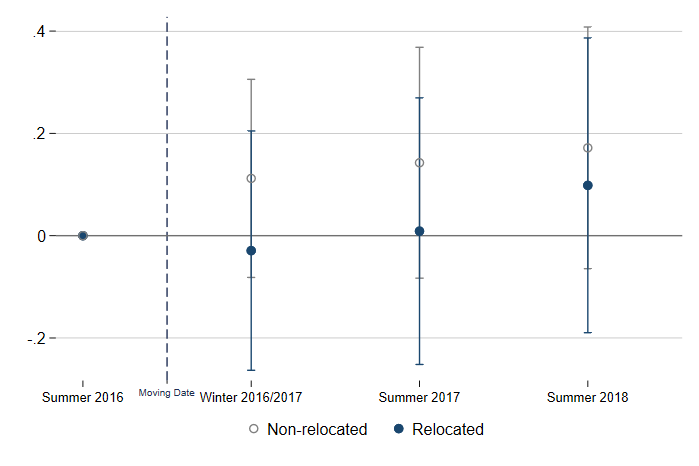

Supplement: S1 File — (ZIP) [file pone.0236029.s002.zip › 03_graphs/dynamic_Q47_3.png]

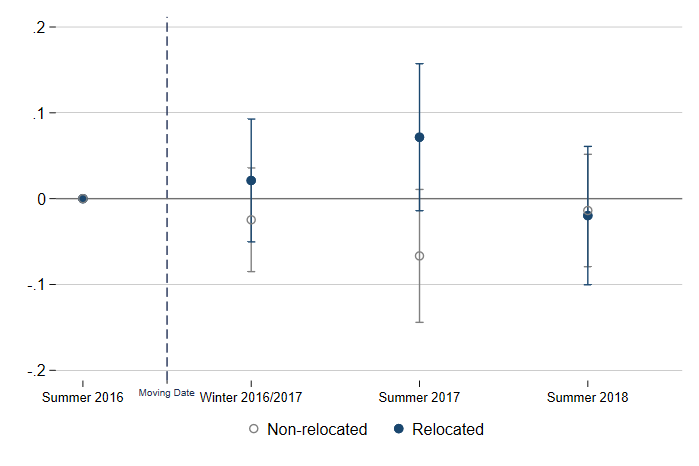

Supplement: S1 File — (ZIP) [file pone.0236029.s002.zip › 03_graphs/dynamic_Q47_5_dm.png]

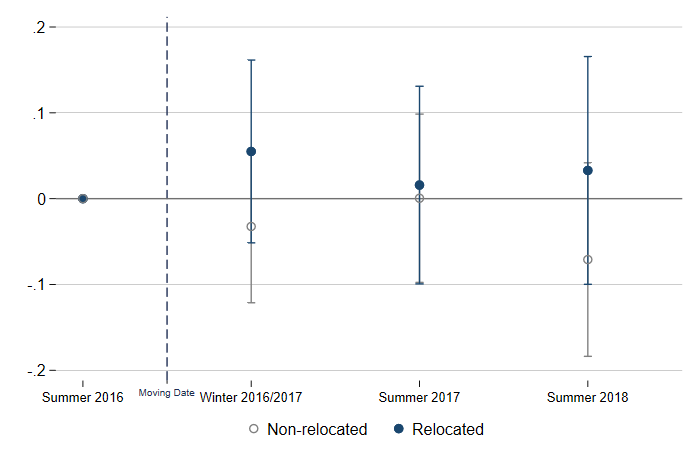

Supplement: S1 File — (ZIP) [file pone.0236029.s002.zip › 03_graphs/dynamic_Q47_10_dm.png]

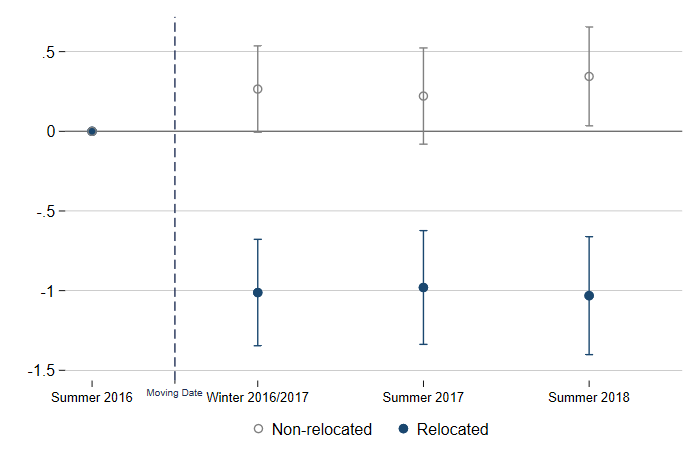

Supplement: S1 File — (ZIP) [file pone.0236029.s002.zip › 03_graphs/dynamic_light_sc_1.png]

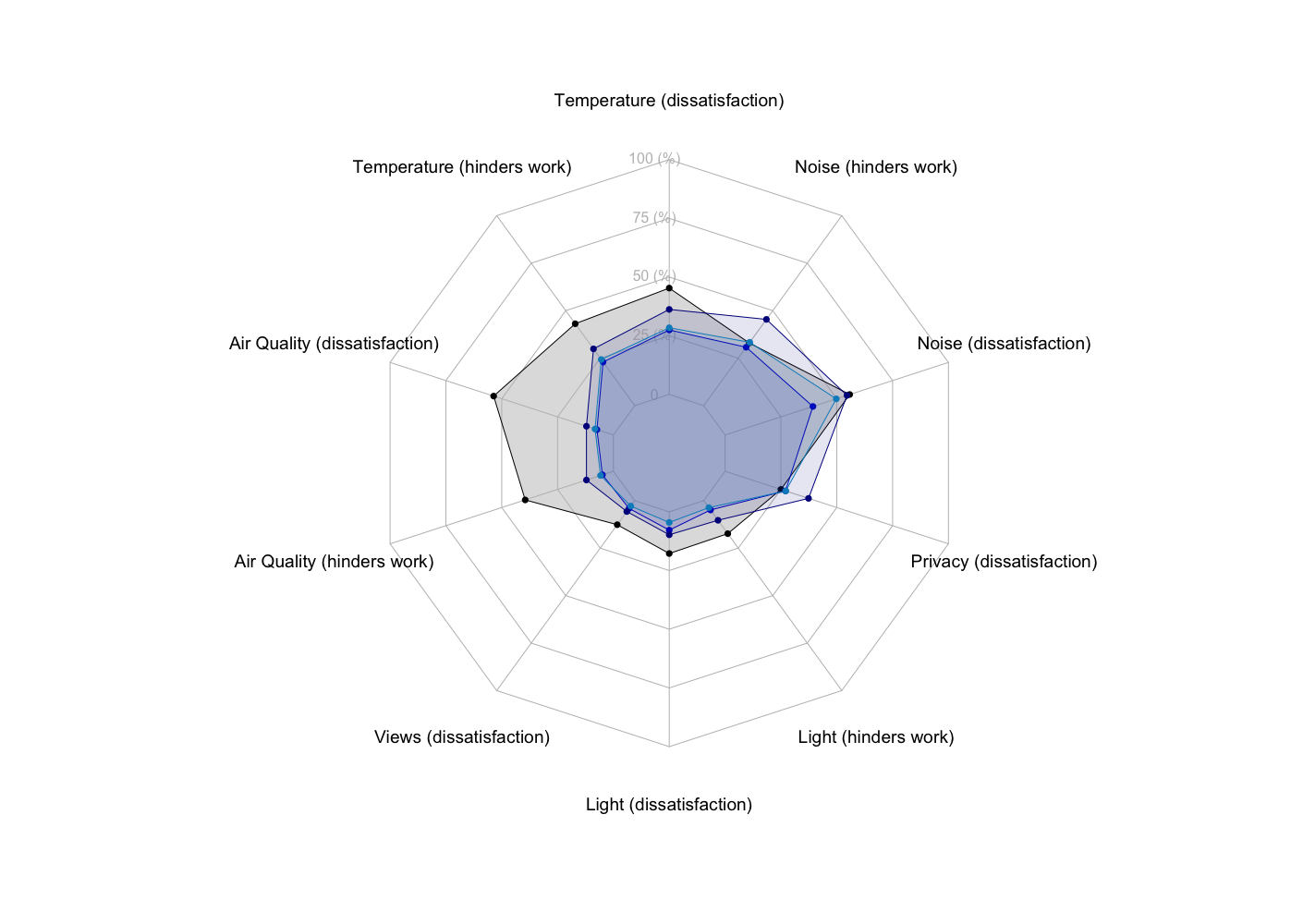

Supplement: S1 File — (ZIP) [file pone.0236029.s002.zip › 03_graphs/radar_treat.tiff]

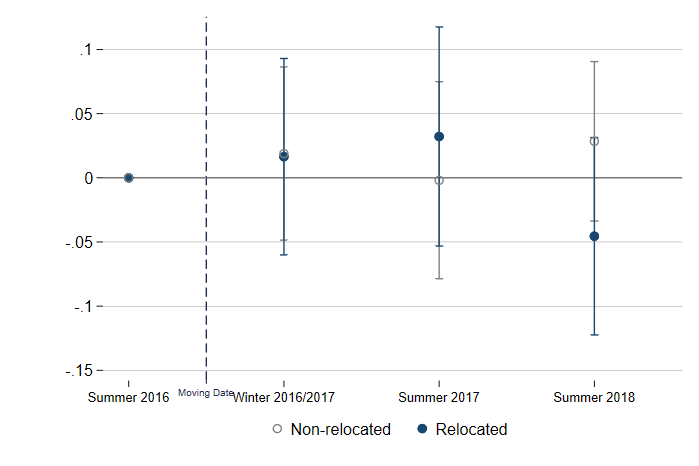

Supplement: S1 File — (ZIP) [file pone.0236029.s002.zip › 03_graphs/dynamic_Q47_1_dm.png]

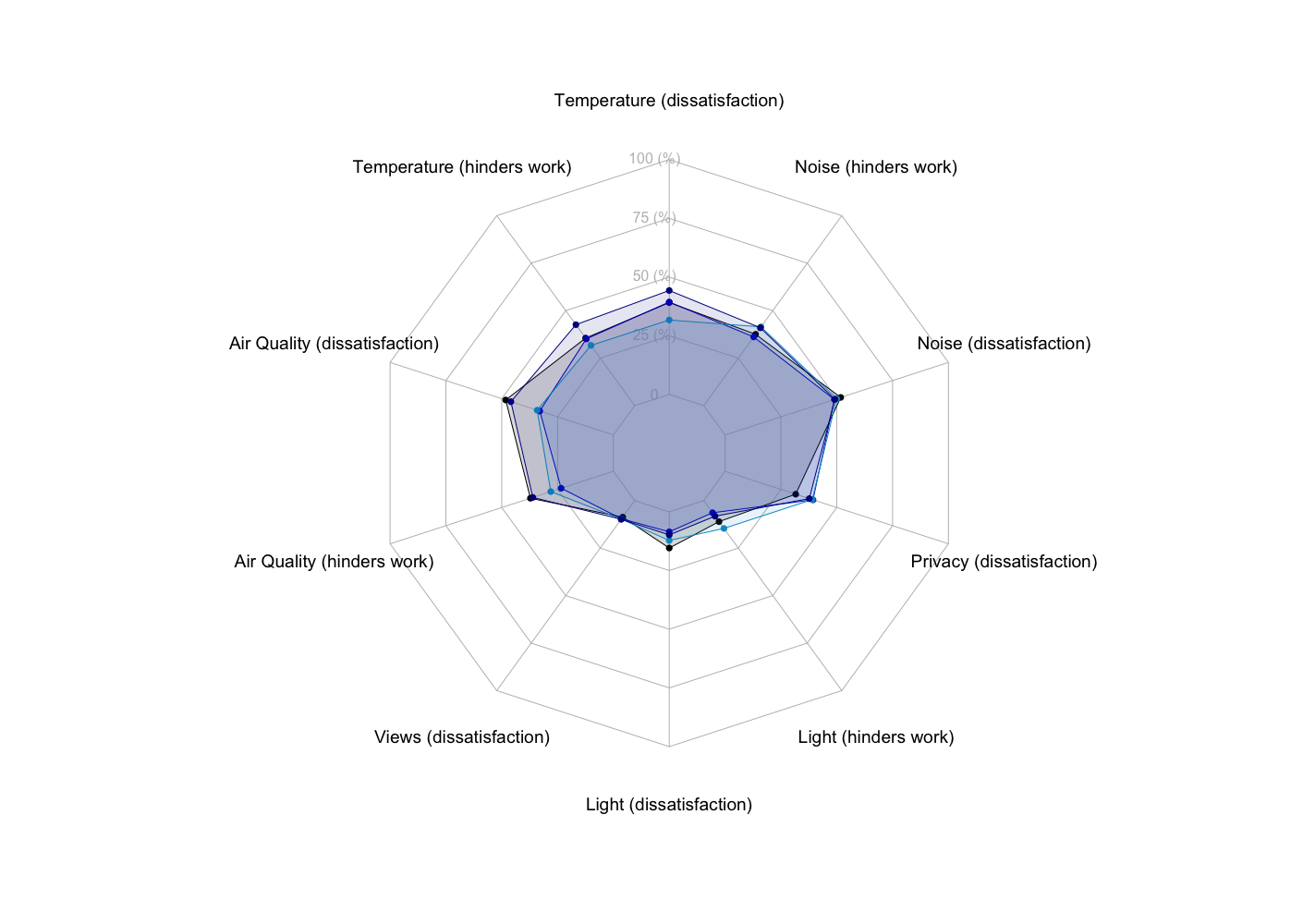

Supplement: S1 File — (ZIP) [file pone.0236029.s002.zip › 03_graphs/radar_control.png]

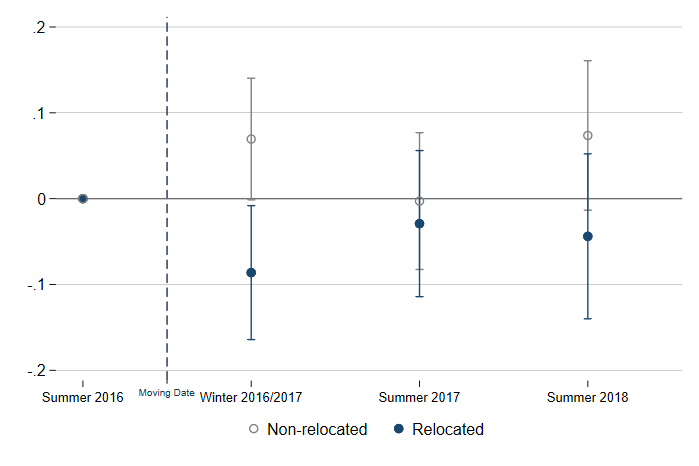

Supplement: S1 File — (ZIP) [file pone.0236029.s002.zip › 03_graphs/dynamic_Q49_6_dm.png]

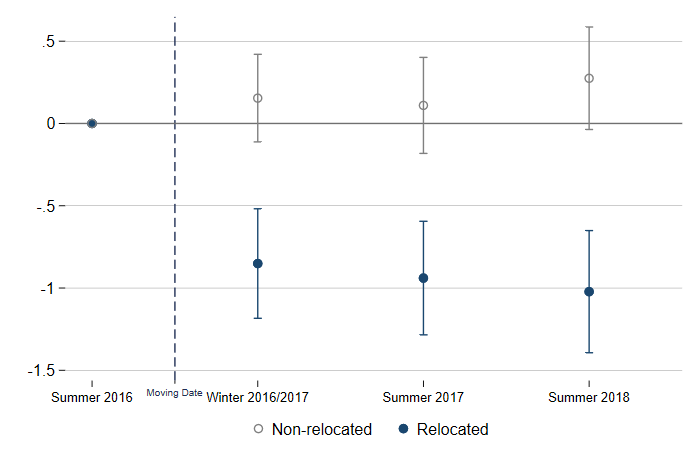

Supplement: S1 File — (ZIP) [file pone.0236029.s002.zip › 03_graphs/dynamic_light_sc_3.png]

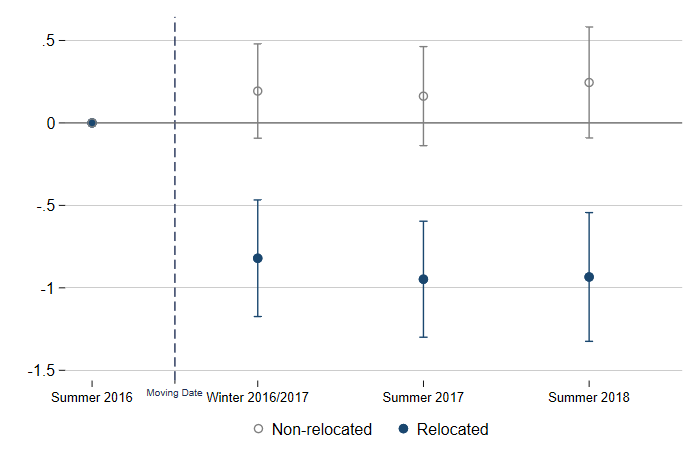

Supplement: S1 File — (ZIP) [file pone.0236029.s002.zip › 03_graphs/dynamic_light_sc_2.png]

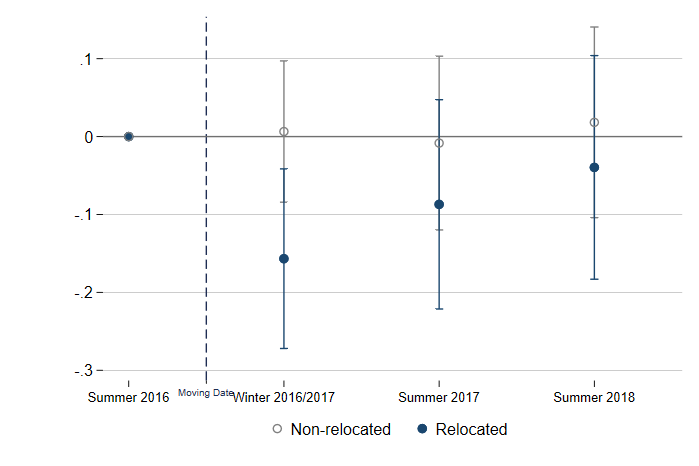

Supplement: S1 File — (ZIP) [file pone.0236029.s002.zip › 03_graphs/dynamic_noise_dm_2.png]

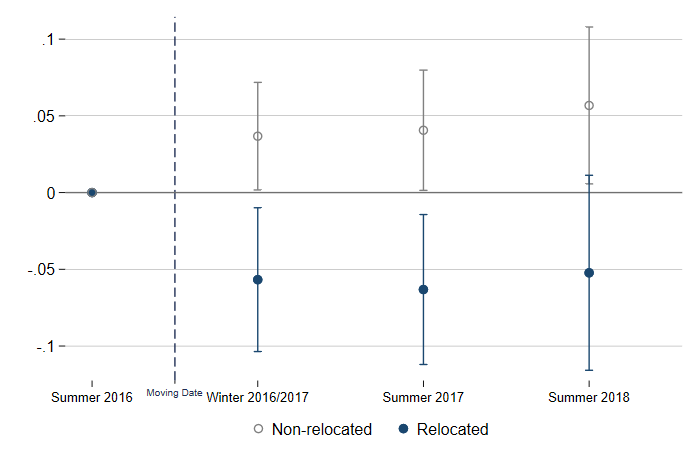

Supplement: S1 File — (ZIP) [file pone.0236029.s002.zip › 03_graphs/dynamic_Q49_4_dm.png]

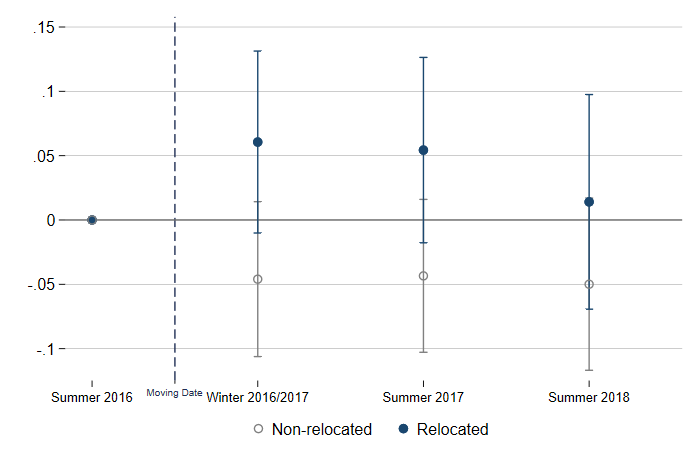

Supplement: S1 File — (ZIP) [file pone.0236029.s002.zip › 03_graphs/dynamic_Q47_3_dm.png]

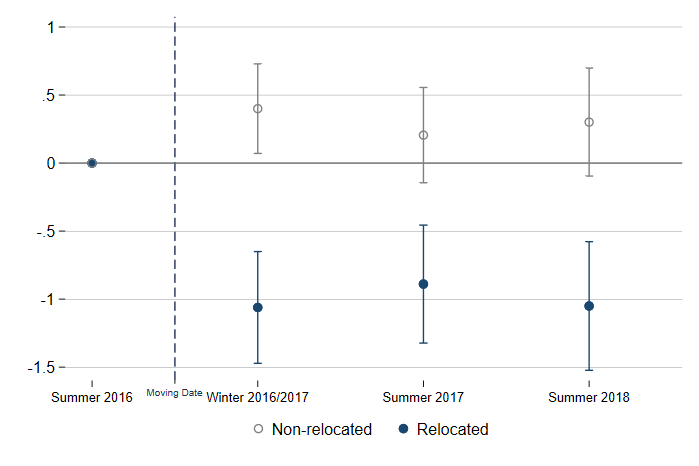

Supplement: S1 File — (ZIP) [file pone.0236029.s002.zip › 03_graphs/dynamic_temp_sc_1.png]

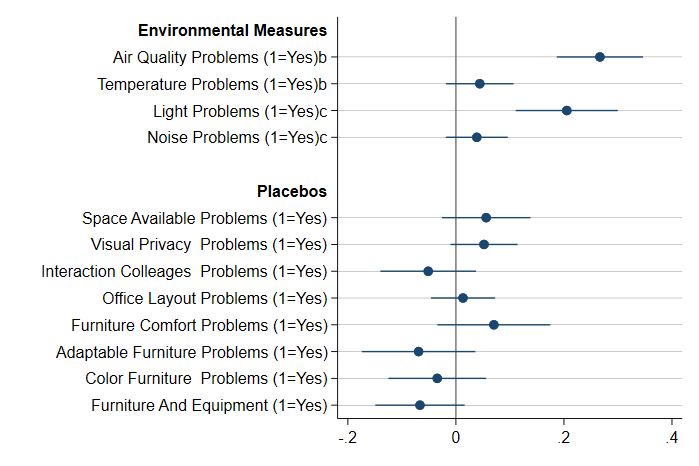

Supplement: S1 File — (ZIP) [file pone.0236029.s002.zip › 03_graphs/sbs_effect_dm_2.png]

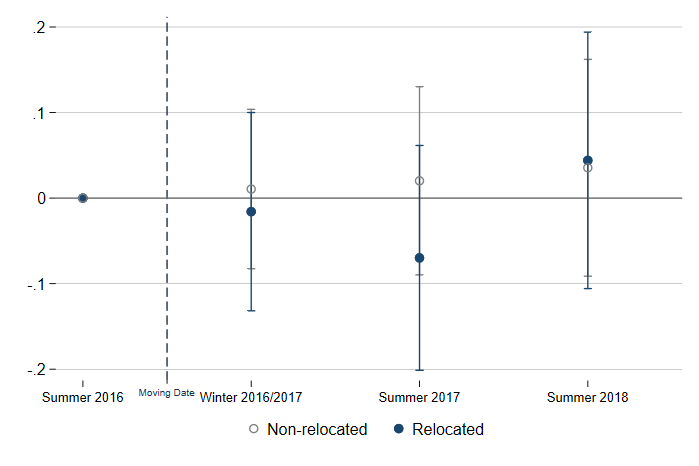

Supplement: S1 File — (ZIP) [file pone.0236029.s002.zip › 03_graphs/dynamic_noise_dm_3.png]

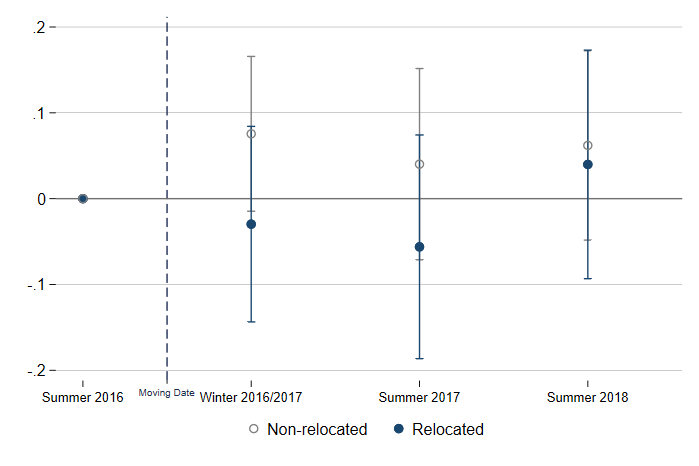

Supplement: S1 File — (ZIP) [file pone.0236029.s002.zip › 03_graphs/dynamic_noise_dm_1.png]

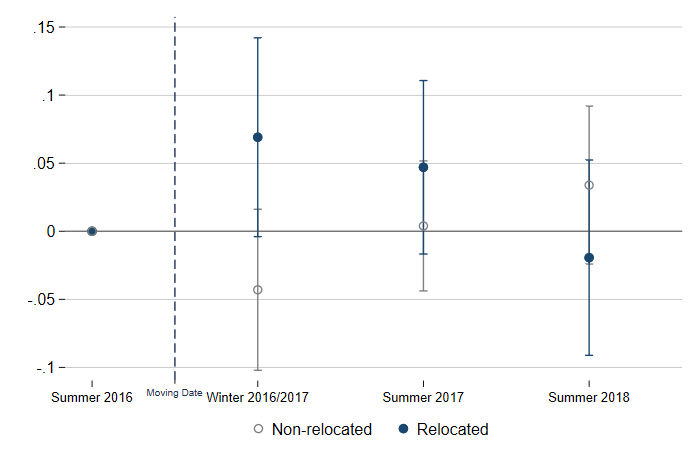

Supplement: S1 File — (ZIP) [file pone.0236029.s002.zip › 03_graphs/dynamic_Q47_12_dm.png]

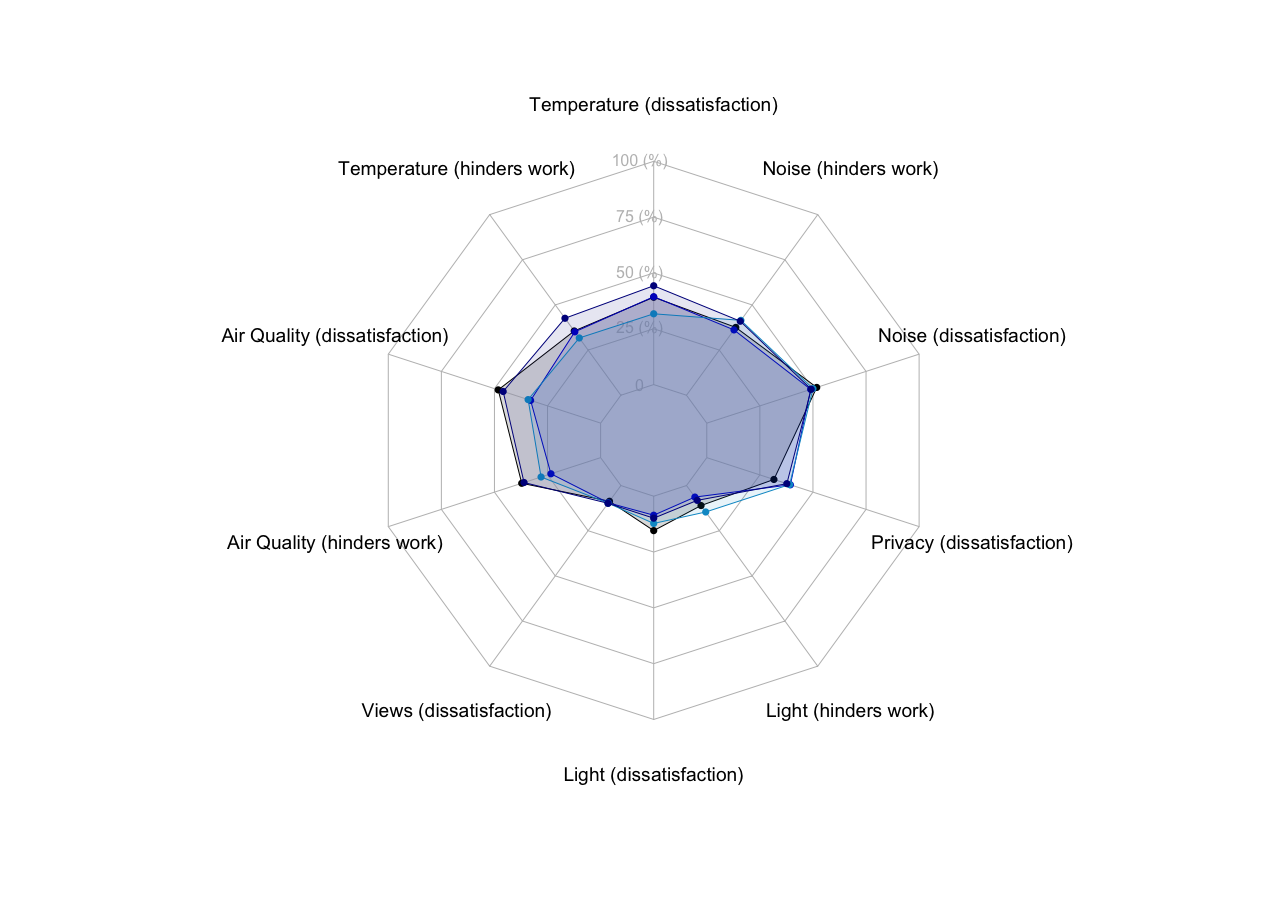

Supplement: S1 File — (ZIP) [file pone.0236029.s002.zip › 03_graphs/radar_control.tiff]

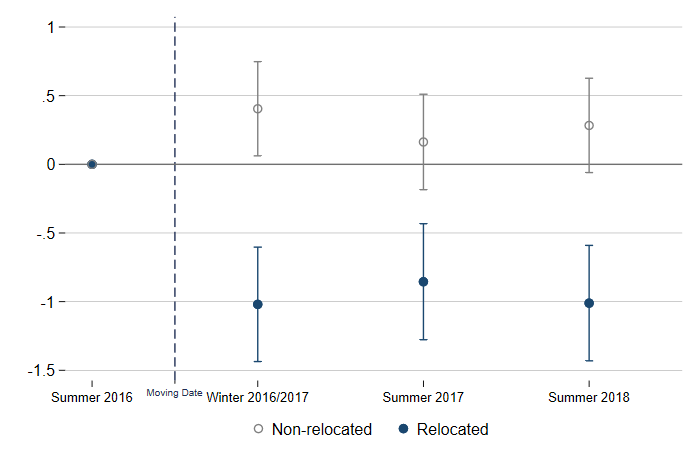

Supplement: S1 File — (ZIP) [file pone.0236029.s002.zip › 03_graphs/dynamic_temp_sc_2.png]

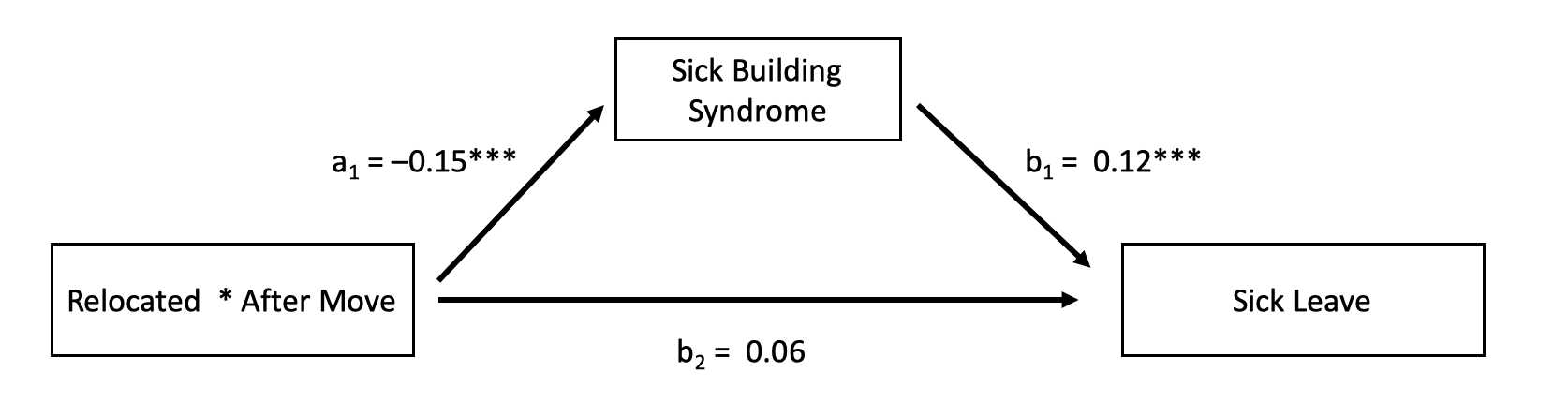

Supplement: S1 File — (ZIP) [file pone.0236029.s002.zip › 03_graphs/mediation_graph_sl.png]

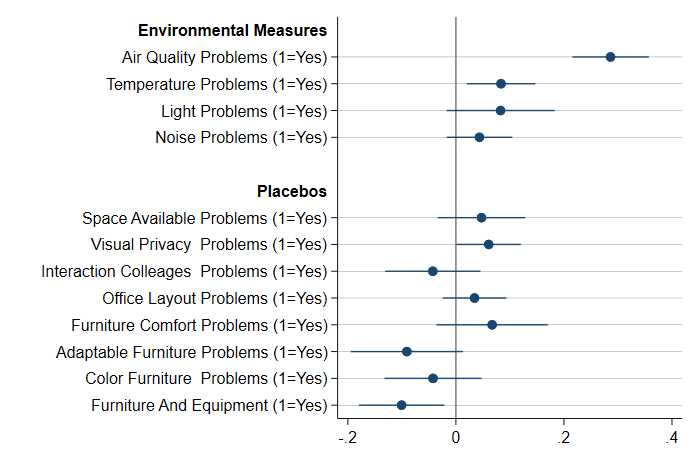

Supplement: S1 File — (ZIP) [file pone.0236029.s002.zip › 03_graphs/sbs_effect_dm_1.png]

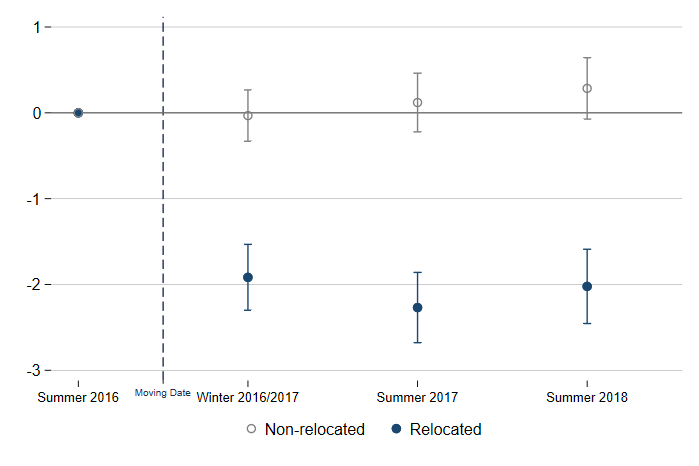

Supplement: S1 File — (ZIP) [file pone.0236029.s002.zip › 03_graphs/dynamic_aq_sc_1.png]

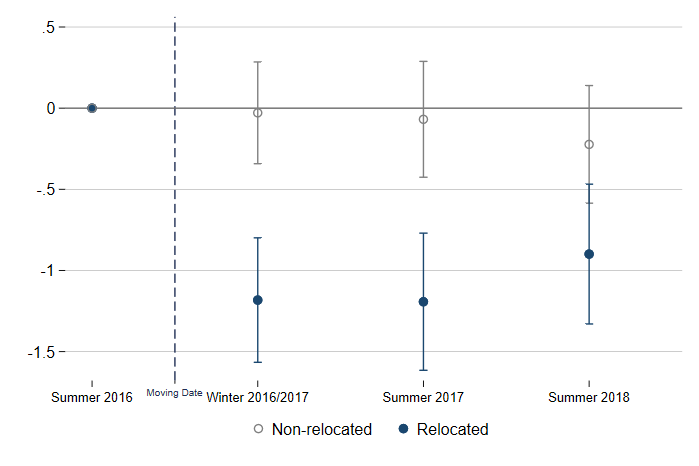

Supplement: S1 File — (ZIP) [file pone.0236029.s002.zip › 03_graphs/dynamic_furniture_sc_1.png]

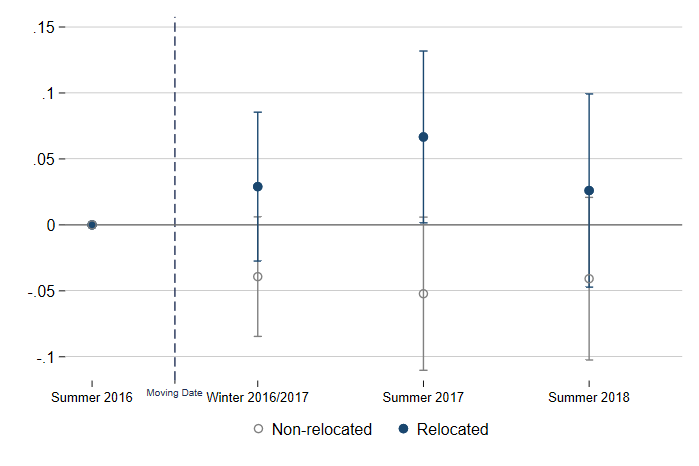

Supplement: S1 File — (ZIP) [file pone.0236029.s002.zip › 03_graphs/dynamic_Q47_6_dm.png]

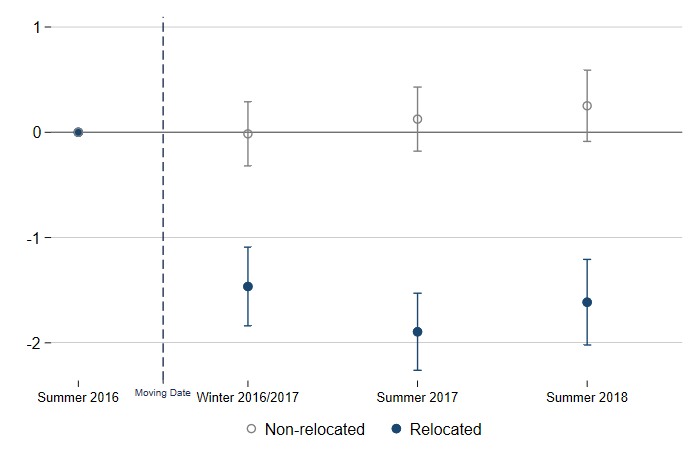

Supplement: S1 File — (ZIP) [file pone.0236029.s002.zip › 03_graphs/dynamic_aq_sc_2.png]

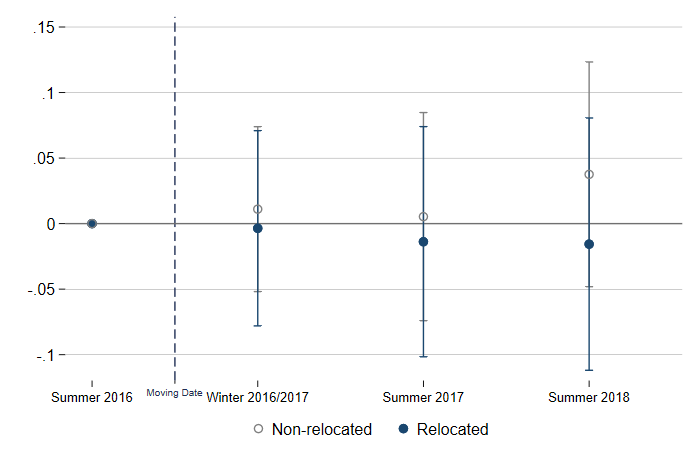

Supplement: S1 File — (ZIP) [file pone.0236029.s002.zip › 03_graphs/dynamic_Q49_1_dm.png]

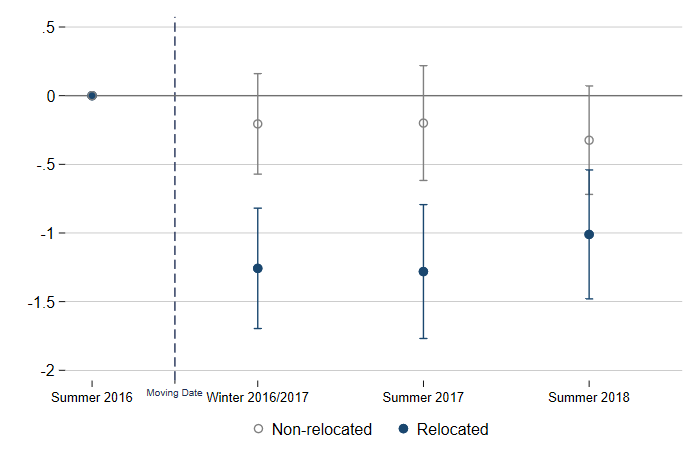

Supplement: S1 File — (ZIP) [file pone.0236029.s002.zip › 03_graphs/dynamic_furniture_sc_2.png]

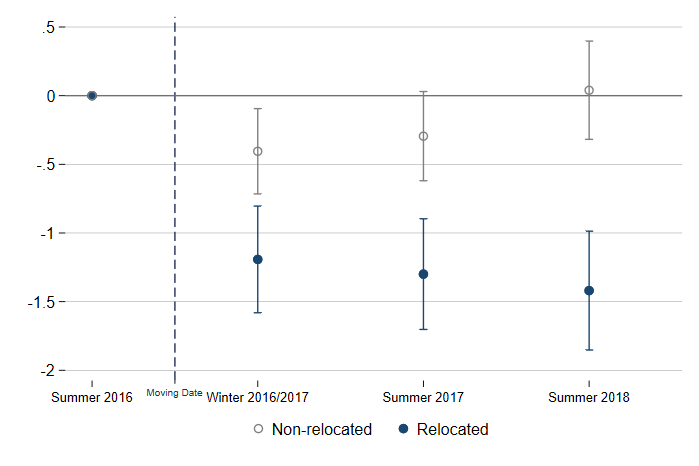

Supplement: S1 File — (ZIP) [file pone.0236029.s002.zip › 03_graphs/dynamic_furniture_sc_3.png]

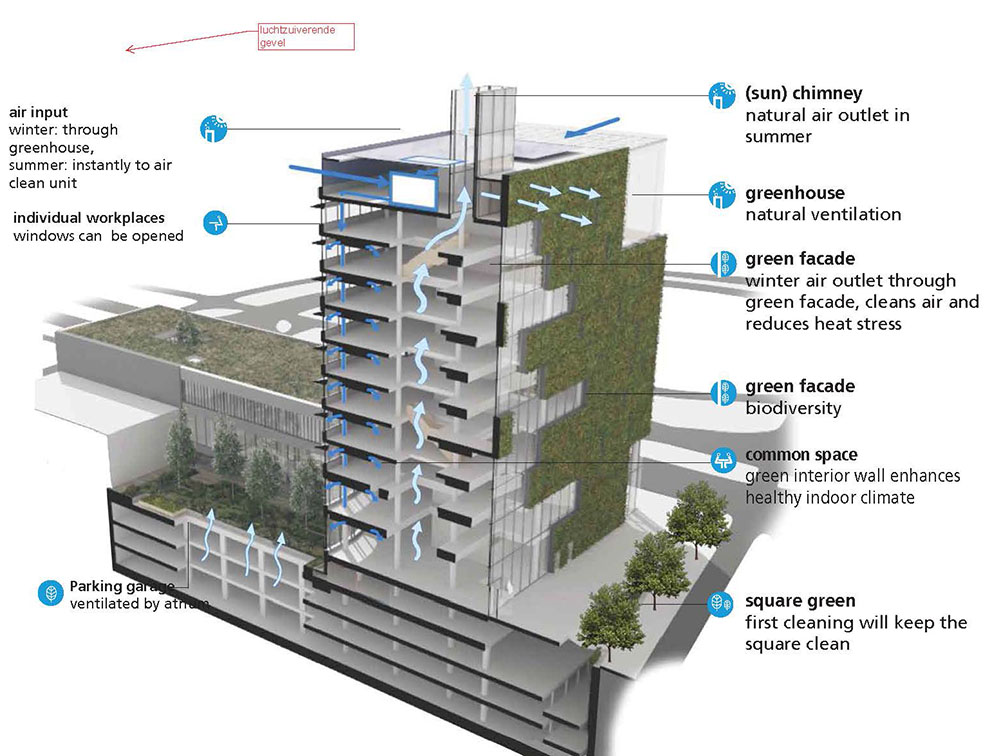

Supplement: S1 File — (ZIP) [file pone.0236029.s002.zip › 03_graphs/diagram_ventilation_building.jpg]

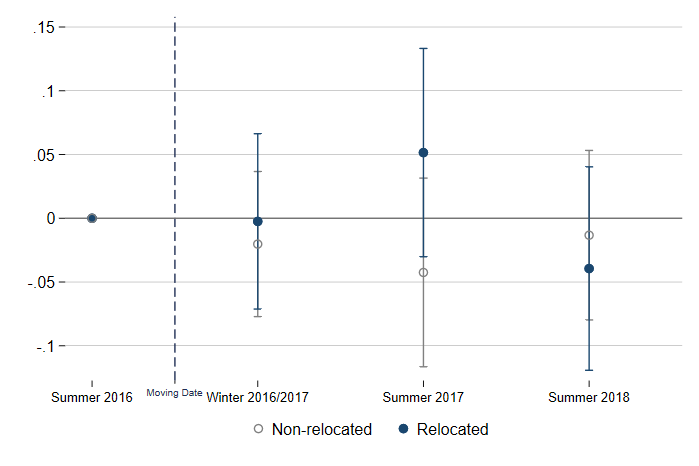

Supplement: S1 File — (ZIP) [file pone.0236029.s002.zip › 03_graphs/dynamic_Q47_8_dm.png]

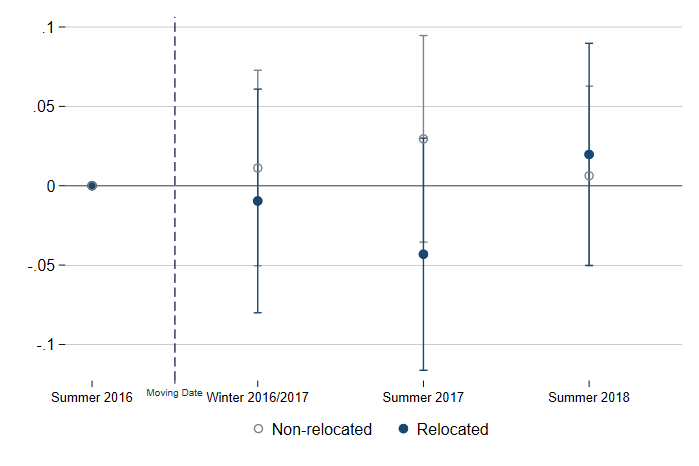

Supplement: S1 File — (ZIP) [file pone.0236029.s002.zip › 03_graphs/dynamic_Q49_3_dm.png]

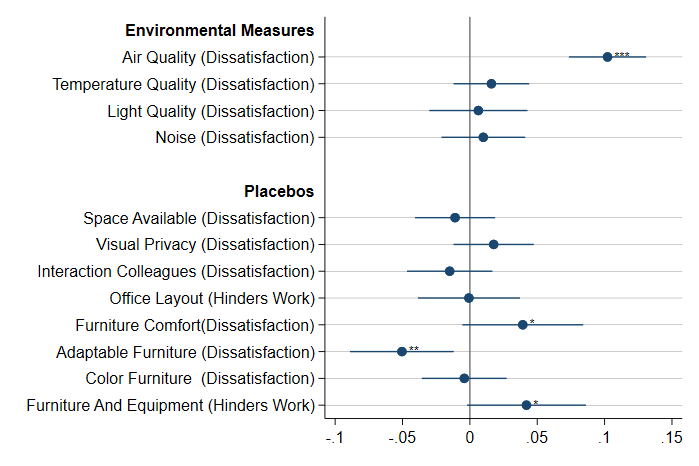

Supplement: S1 File — (ZIP) [file pone.0236029.s002.zip › 03_graphs/sbs_effect_sc_1tt.png]

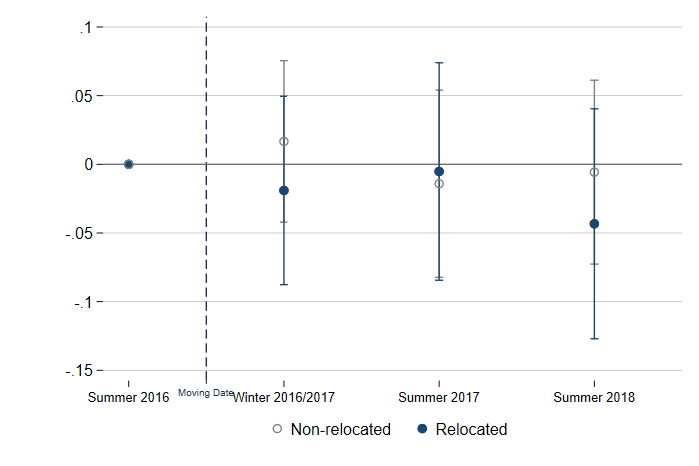

Supplement: S1 File — (ZIP) [file pone.0236029.s002.zip › 03_graphs/dynamic_Q47_4_dm.png]

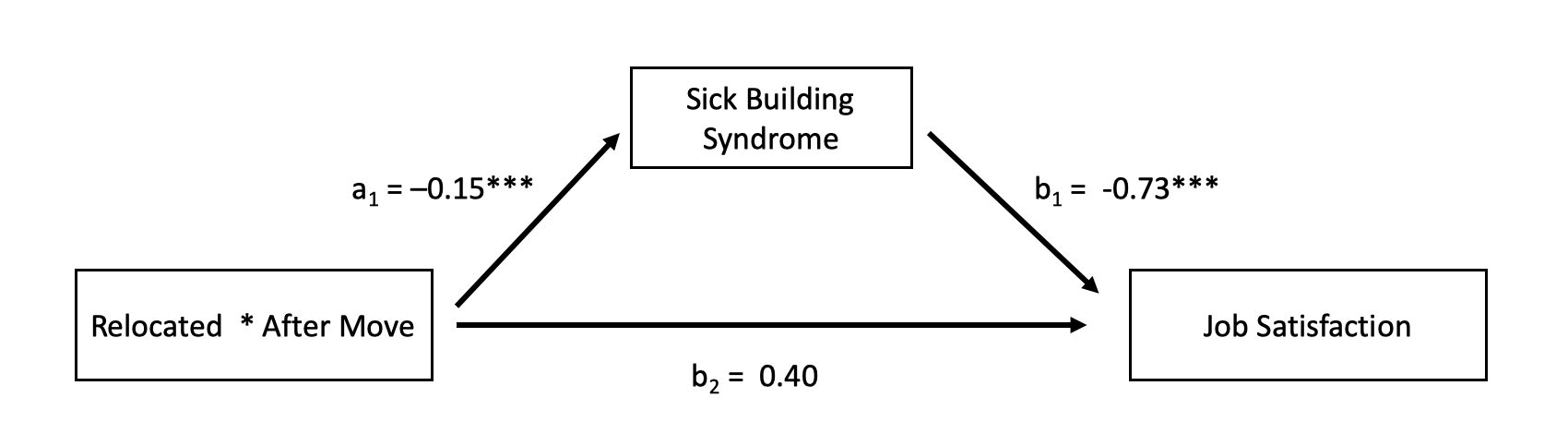

Supplement: S1 File — (ZIP) [file pone.0236029.s002.zip › 03_graphs/mediation_graph_js.png]

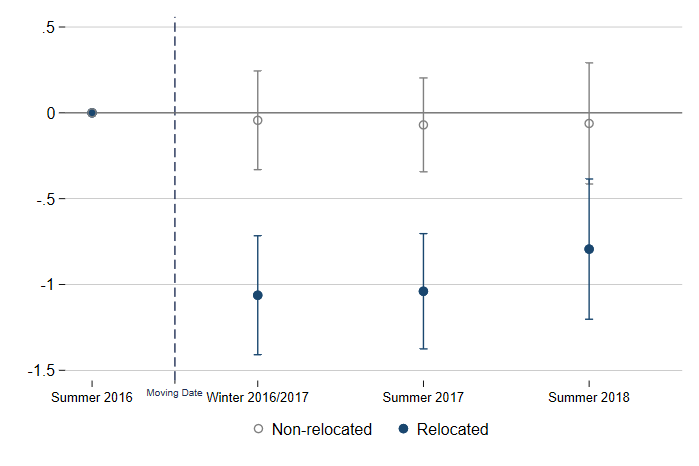

Supplement: S1 File — (ZIP) [file pone.0236029.s002.zip › 03_graphs/dynamic_furniture_sc_4.png]

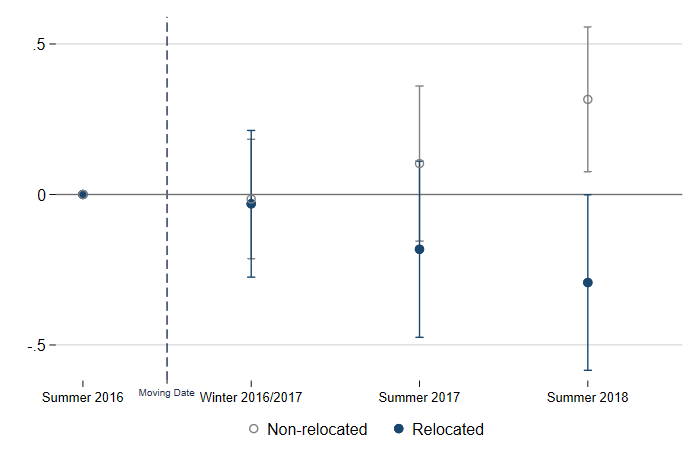

Supplement: S1 File — (ZIP) [file pone.0236029.s002.zip › 03_graphs/dynamic_Q49_1.png]

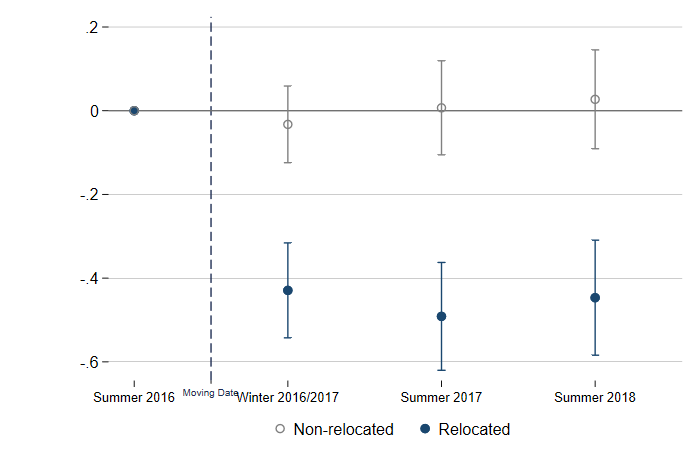

Supplement: S1 File — (ZIP) [file pone.0236029.s002.zip › 03_graphs/dynamic_aq_dm_1.png]

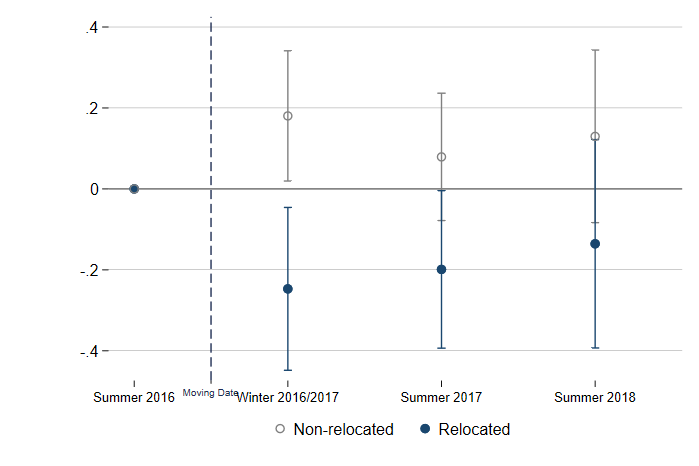

Supplement: S1 File — (ZIP) [file pone.0236029.s002.zip › 03_graphs/dynamic_Q49_2.png]

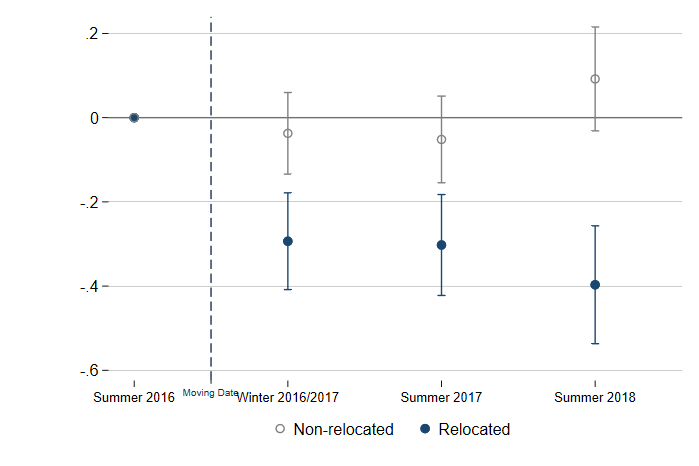

Supplement: S1 File — (ZIP) [file pone.0236029.s002.zip › 03_graphs/dynamic_aq_dm_2.png]

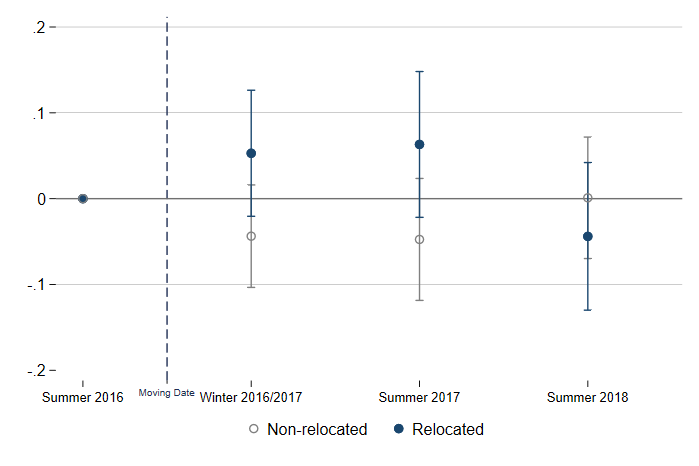

Supplement: S1 File — (ZIP) [file pone.0236029.s002.zip › 03_graphs/dynamic_Q47_11_dm.png]

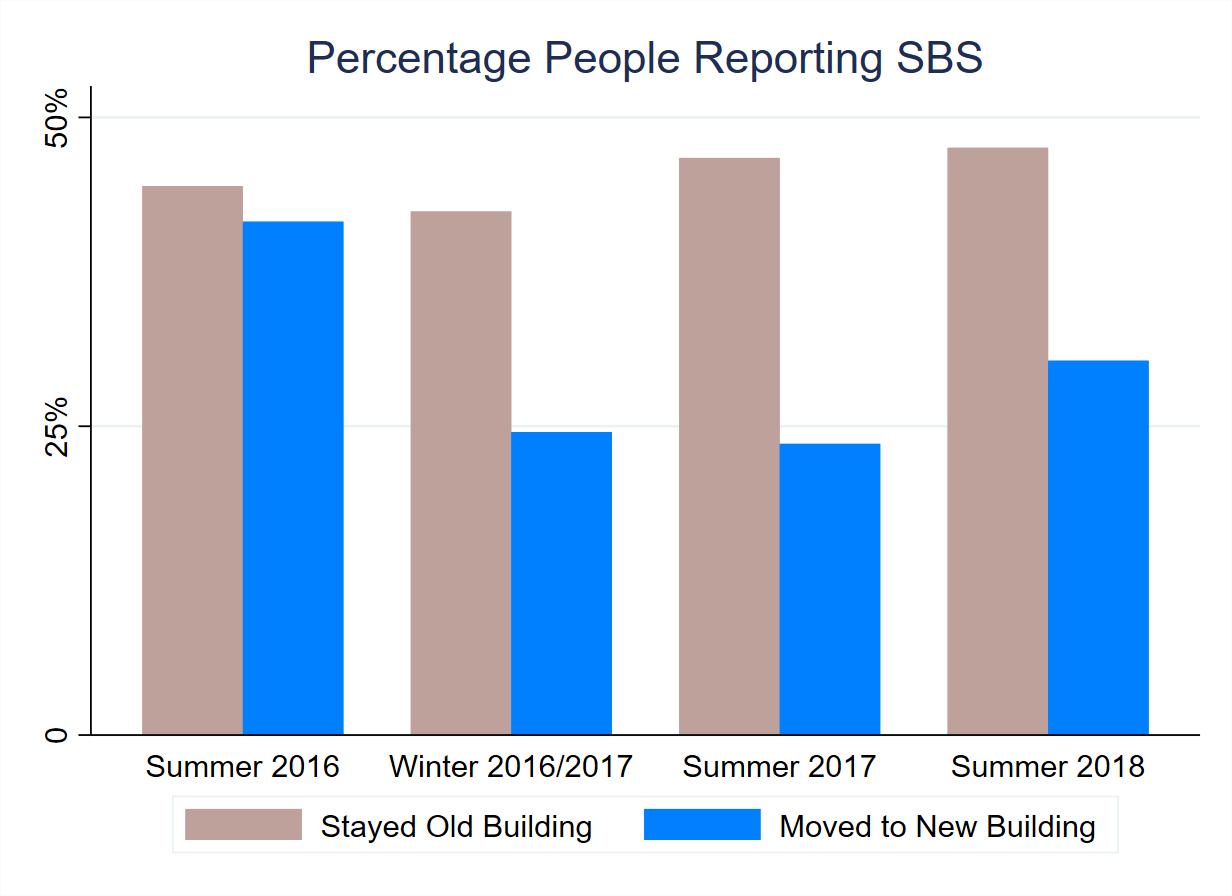

Supplement: S1 File — (ZIP) [file pone.0236029.s002.zip › 03_graphs/sbs_bar.png]

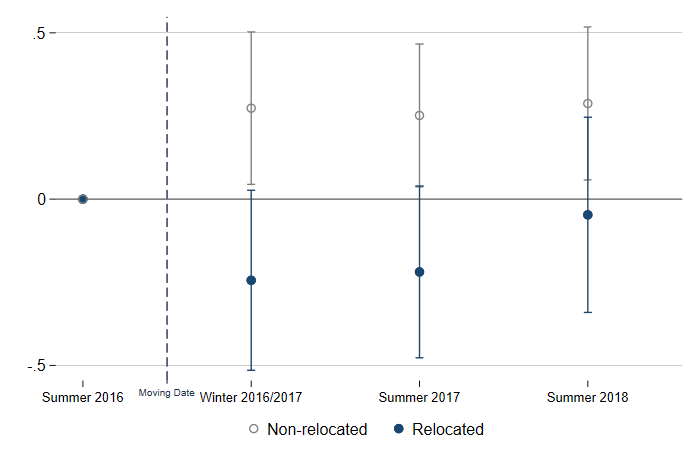

Supplement: S1 File — (ZIP) [file pone.0236029.s002.zip › 03_graphs/dynamic_Q49_3.png]

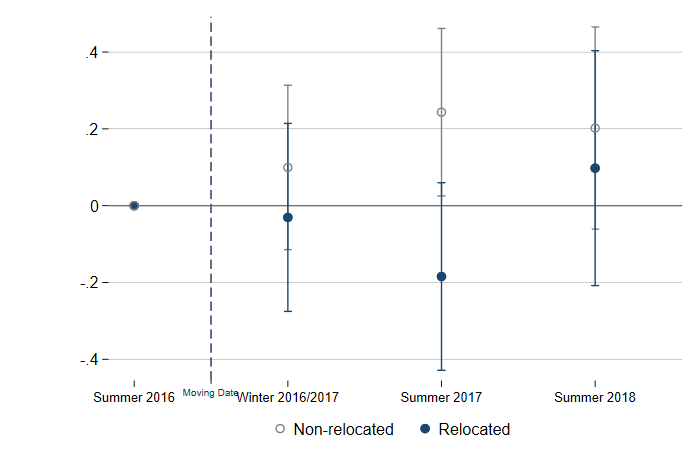

Supplement: S1 File — (ZIP) [file pone.0236029.s002.zip › 03_graphs/dynamic_Q47_8.png]

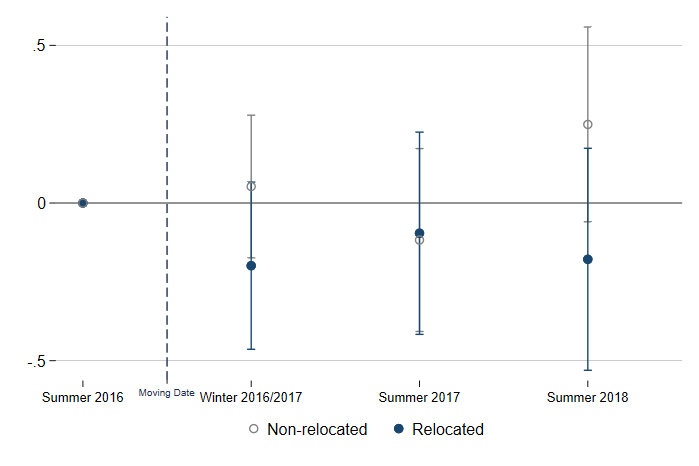

Supplement: S1 File — (ZIP) [file pone.0236029.s002.zip › 03_graphs/dynamic_Q49_6.png]

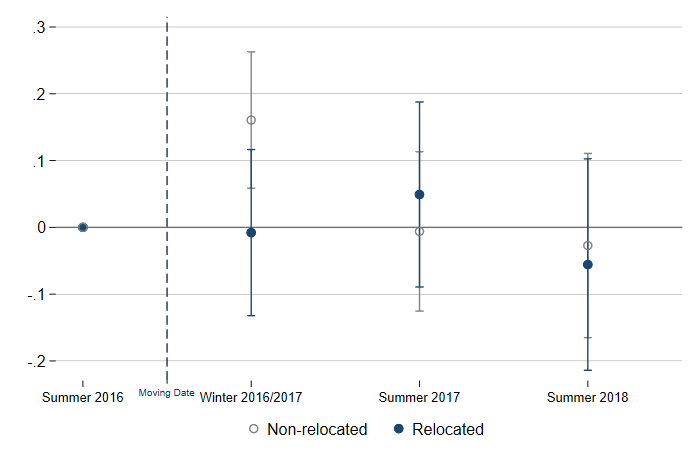

Supplement: S1 File — (ZIP) [file pone.0236029.s002.zip › 03_graphs/dynamic_no_days_sick.png]

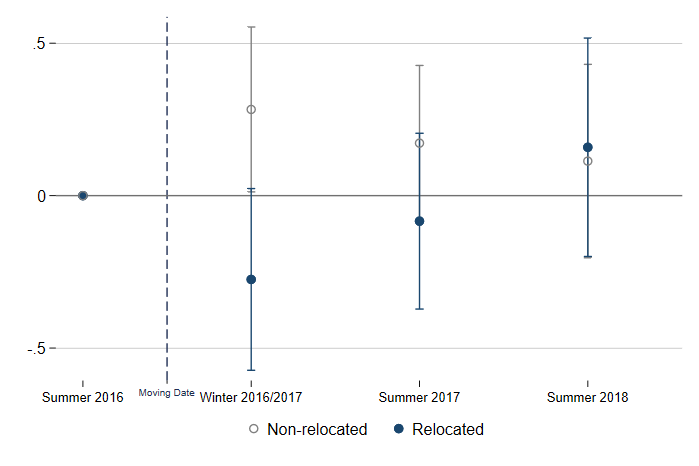

Supplement: S1 File — (ZIP) [file pone.0236029.s002.zip › 03_graphs/dynamic_Q47_9.png]

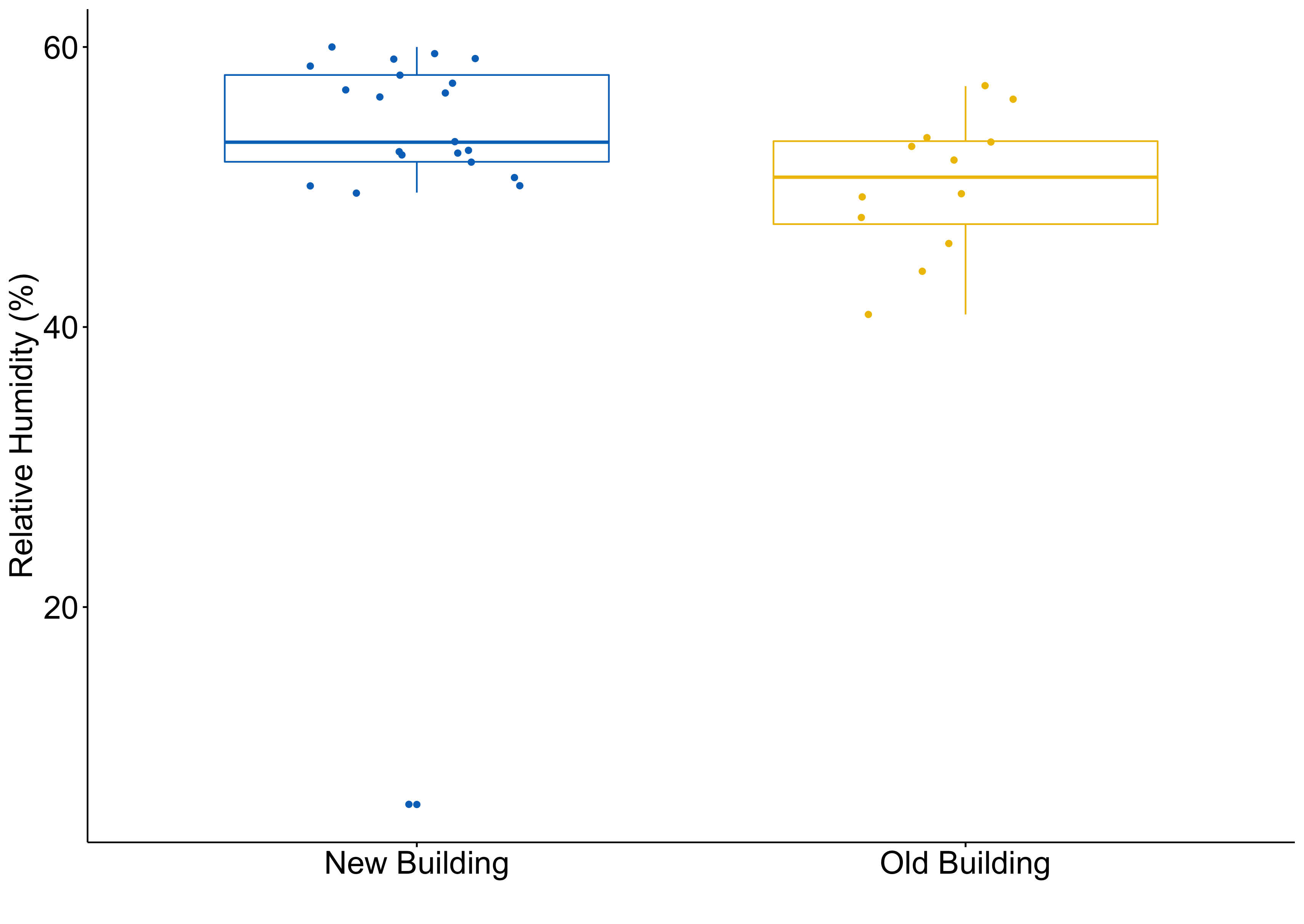

Supplement: S1 File — (ZIP) [file pone.0236029.s002.zip › 03_graphs/meas_rh.png]

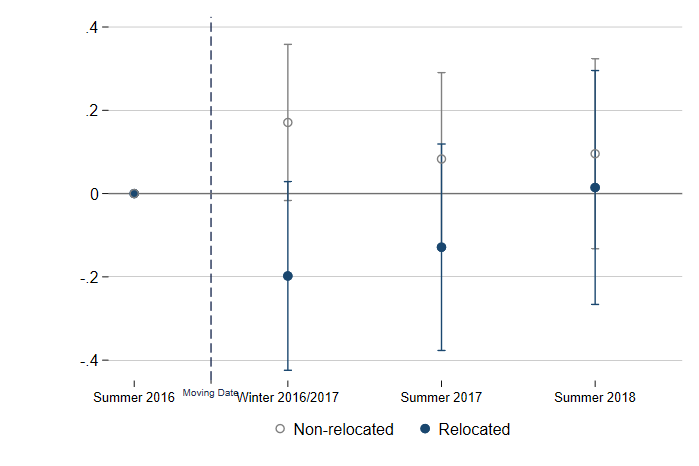

Supplement: S1 File — (ZIP) [file pone.0236029.s002.zip › 03_graphs/dynamic_Q49_4.png]

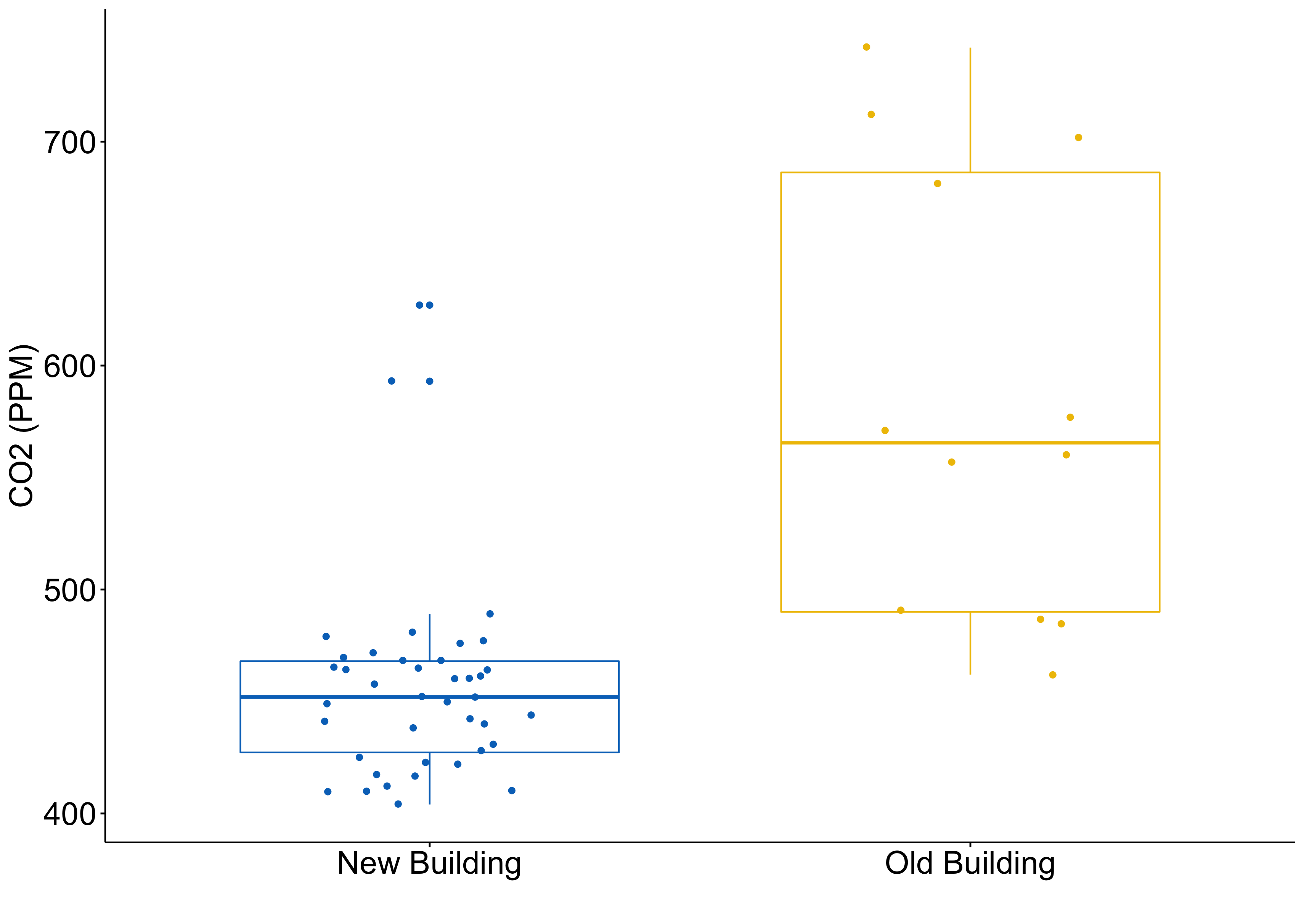

Supplement: S1 File — (ZIP) [file pone.0236029.s002.zip › 03_graphs/meas_co2.png]

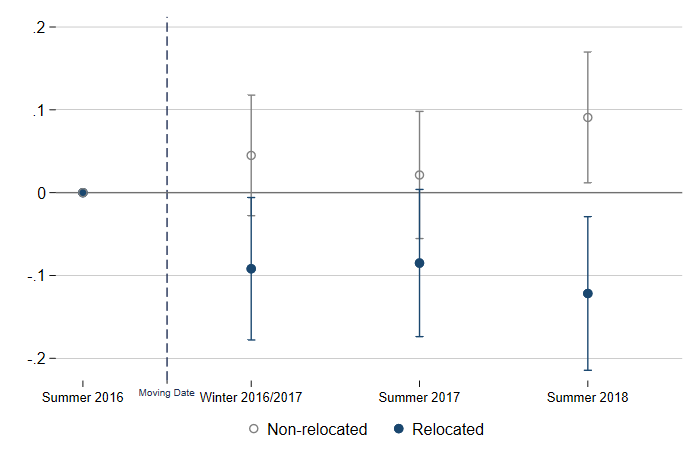

Supplement: S1 File — (ZIP) [file pone.0236029.s002.zip › 03_graphs/dynamic_Q49_5_dm.png]

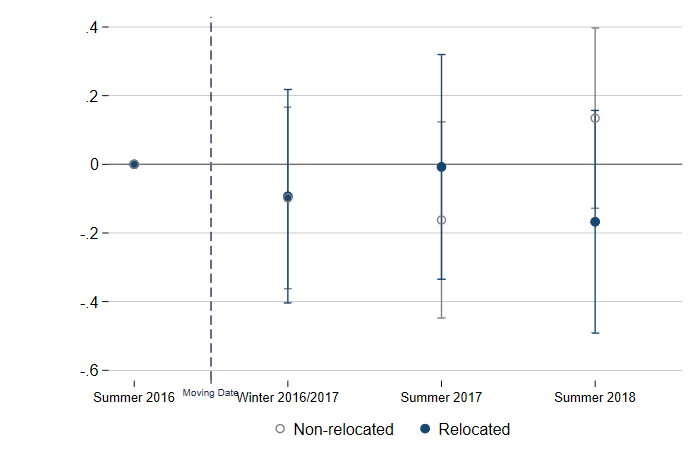

Supplement: S1 File — (ZIP) [file pone.0236029.s002.zip › 03_graphs/dynamic_Q49_5.png]

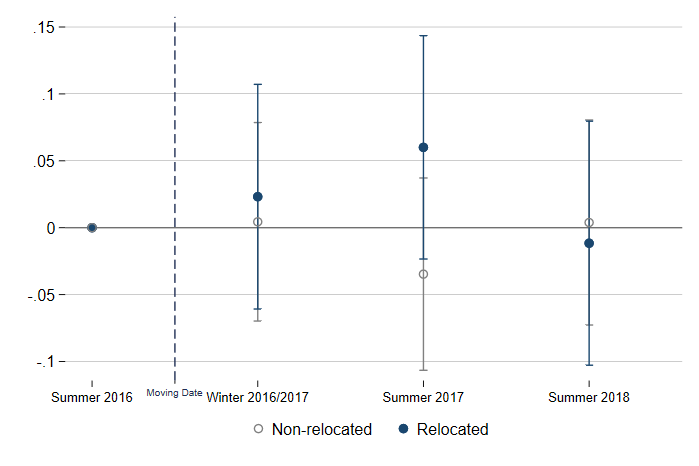

Supplement: S1 File — (ZIP) [file pone.0236029.s002.zip › 03_graphs/dynamic_Q47_2_dm.png]
