# Supplementary material for: Moving to productivity: The benefits of healthy buildings
Source: PLoS One. 2020 Aug 6;15(8):e0236029. doi: 10.1371/journal.pone.0236029 (PMC7410200; doi:10.1371/journal.pone.0236029)
Supplement: S2 File — (ZIP) [file pone.0236029.s003.zip › 03_tables/diff.pdf]

** *********
****
*** ** ***
****** **
** *********
** *** **
*** ** *** **
