# Supplementary material for: Moving to productivity: The benefits of healthy buildings
Source: PLoS One. 2020 Aug 6;15(8):e0236029. doi: 10.1371/journal.pone.0236029 (PMC7410200; doi:10.1371/journal.pone.0236029)
Supplement: S2 File — (ZIP) [file pone.0236029.s003.zip › 03_tables/document_v1.pdf]

Moving to Productivity: The Benefits of Healthy Buildings

Piet Eichholtz* Nils Kok† Juan Palacios‡

Preliminary Working Paper
April 6, 2019

*Maastricht University, The Netherlands; p.eichholtz@maastrichtuniversity.nl; Corresponding address: Maastricht University, School of Business and Economics, Department of Economics, Tongersestraat 53, 6211 LM Maastricht, The Netherlands. Fax: +31 433 882000.

†Maastricht University, The Netherlands

‡Maastricht University, The Netherlands; IZA Bonn, Germany

1 Empirical Strategy

We use difference-in-difference (DiD) models to estimate the impact of the improvement in building condions

Table 1: Descriptive statistics sample before the move

	Control (N=247)	Treated (N=326)	Differences
Age			
Below 31 years old (1=Yes)	0.19	0.10	0.09**
31-50 year old (1=Yes)	0.34	0.45	-0.11**
50 years old or older (1=Yes)	0.47	0.45	0.02
Gender			
Female (1=Yes)	0.46	0.50	-0.04
Health			
Sick Building Syndrome (1=Yes)	0.44	0.42	0.03
No days on sick leave (1=yes)	0.53	0.53	-0.01
Time Working for The Company			
Less than 1 year	0.23	0.12	0.11***
1-2 years	0.38	0.24	0.14***
3-5 years	0.16	0.27	-0.11**
More than 5 years	0.23	0.37	-0.14***
Working Hours per Week			
Less than 10 hours	0.06	0.03	0.03
11-30 hours	0.41	0.49	-0.08
More than 30 hours	0.53	0.48	0.05

* p<0.05, ** p<0.01, *** p<0.001

Table 2: Basic difference-in-difference results

	Before Moving Date			After Moving Date		
	Control	Treat.	Diff	Control	Treat.	Diff
Indoor Environmental Conditions						
Air Quality (Dissatisfaction)	4.18	4.50	-0.31**	3.95	2.65	1.30***
Air Quality (Hinders Work)	3.95	4.14	-0.19	3.80	2.68	1.12***
Temperature Quality (Dissatisfaction)	3.94	4.15	-0.21	4.07	3.60	0.47***
Temperature Quality (Hinders Work)	3.84	4.02	-0.18	3.92	3.45	0.47***
Light Quality (Dissatisfaction)	2.57	2.83	-0.26**	2.68	2.15	0.53***
Views (Dissatisfaction)	2.83	3.14	-0.31**	2.85	2.50	0.34***
Light Quality (Hinders Work)	2.90	3.25	-0.35***	2.93	2.58	0.35***
Noise (Dissatisfaction)	3.61	3.58	0.03	3.73	3.82	-0.10
Privacy (Dissatisfaction)	4.40	4.54	-0.15	4.41	4.37	0.05
Noise (Hinders Work)	3.93	4.03	-0.10	4.00	4.06	-0.06
Office Layout						
Space Available (Dissatisfaction)	3.03	2.66	0.38***	2.90	2.72	0.19**
Visual Privacy (Dissatisfaction)	3.41	3.06	0.35**	3.51	3.35	0.15
Interaction Colleagues (Dissatisfaction)	2.58	2.48	0.11	2.47	2.43	0.04
Office Layout (Hinders Work)	3.05	2.89	0.16	3.02	2.85	0.16*
Furniture Comfort(Dissatisfaction)	3.18	3.37	-0.19	2.81	2.23	0.58***
Adaptable Furniture (Dissatisfaction)	3.22	3.56	-0.34**	2.77	2.20	0.58***
Color Furniture (Dissatisfaction)	3.68	3.87	-0.19	3.06	2.32	0.73***
Furniture And Equipment (Hinders Work)	3.17	3.27	-0.10	2.89	2.31	0.58***
Health						
Sick Building Syndrome (1=Yes)	0.44	0.42	0.03	0.45	0.26	0.19***
No days on sick leave (1=yes)	0.53	0.53	-0.01	0.61	0.57	0.04

Table 3: Estimation results difference in difference results for different set of fixed effects

	(1) Basic DID	(2) Wave FEs	(3) Indiv.FEs	(4) All FEs	(5) FEs & Controls
Air Quality (Dissatisfaction)	-1.615***	-1.612***	-2.066***	-2.068***	-1.629***
	(0.163)	(0.163)	(0.179)	(0.179)	(0.170)
Air Quality (Hinders Work)	-1.314***	-1.314***	-1.654***	-1.654***	-1.276***
	(0.146)	(0.146)	(0.165)	(0.165)	(0.154)
Temperature Quality (Dissatisfaction)	-0.684***	-0.679***	-1.000***	-0.999***	-0.751***
	(0.162)	(0.163)	(0.191)	(0.192)	(0.191)
Temperature Quality (Hinders Work)	-0.653***	-0.650***	-0.962***	-0.960***	-0.730***
	(0.155)	(0.155)	(0.183)	(0.183)	(0.178)
Light Quality (Dissatisfaction)	-0.796***	-0.794***	-1.006***	-1.006***	-0.643***
	(0.141)	(0.141)	(0.152)	(0.152)	(0.146)
Views (Dissatisfaction)	-0.648***	-0.651***	-0.895***	-0.895***	-0.542***
	(0.139)	(0.139)	(0.153)	(0.153)	(0.142)
Light Quality (Hinders Work)	-0.703***	-0.703***	-0.927***	-0.926***	-0.621***
	(0.137)	(0.136)	(0.152)	(0.153)	(0.142)
Noise (Dissatisfaction)	0.118	0.127	0.004	0.002	0.040
	(0.160)	(0.161)	(0.172)	(0.171)	(0.146)
Privacy (Dissatisfaction)	-0.206	-0.194	-0.336	-0.339	-0.193
	(0.171)	(0.171)	(0.180)	(0.180)	(0.164)
Noise (Hinders Work)	-0.051	-0.042	-0.182	-0.186	-0.070
	(0.148)	(0.149)	(0.162)	(0.162)	(0.136)
Sick Building Syndrome (1=Yes)	-0.165***	-0.164***	-0.286***	-0.286***	-0.268***
	(0.049)	(0.049)	(0.053)	(0.053)	(0.054)
No days on sick leave (1=yes)	-0.033	-0.041	-0.004	-0.001	0.028
	(0.049)	(0.049)	(0.055)	(0.055)	(0.059)

Standard errors in parentheses

* $p < 0.05$, ** $p < 0.01$, *** $p < 0.001$

Figure 1: Trends in Environmental Perception

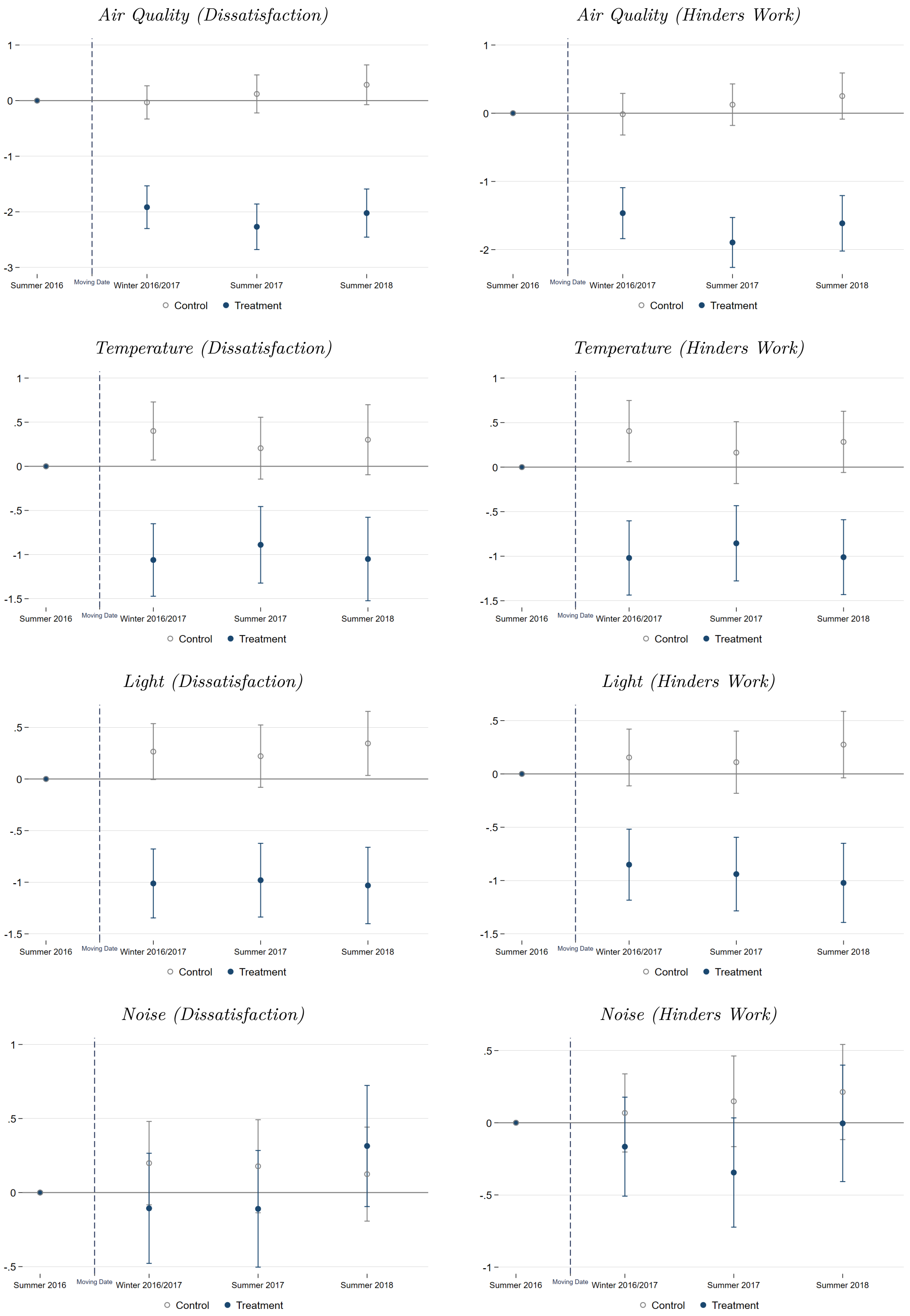

Note: The figure shows the estimated coefficient of the time dummies describing the survey times before (= 0) and after the moving date. The dots represent the point estimates and the bars the 95% confidence intervals. All regressions include the *Elo score* individual fixed effects. The vertical, dashed gray line indicates the moving date.

Figure 2: Trends in Sick Building Syndrome (1=Yes)

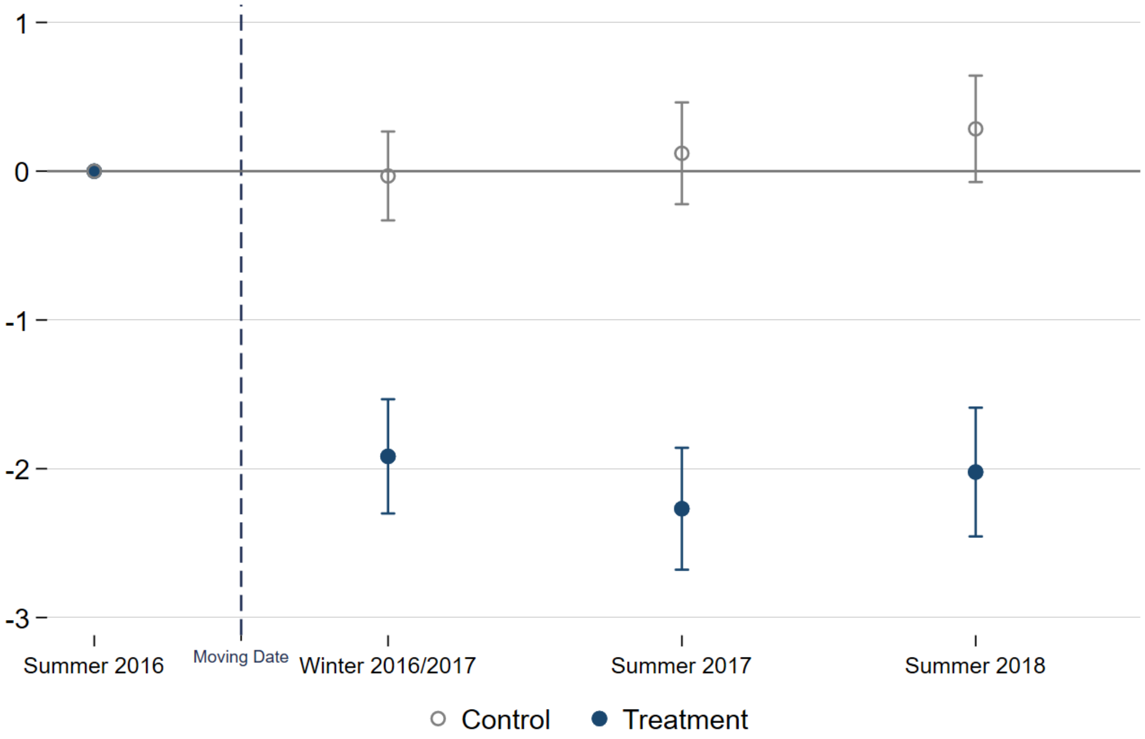

Note: The figure shows the estimated coefficient of the time dummies describing the survey times before (= 0) and after the moving date. The dots represent the point estimates and the bars the 95% confidence intervals. All regressions include the *Elo score* individual fixed effects. The vertical, dashed gray line indicates the moving date.

Figure 3: Effect Environmental Problems on Sick Building Syndrome

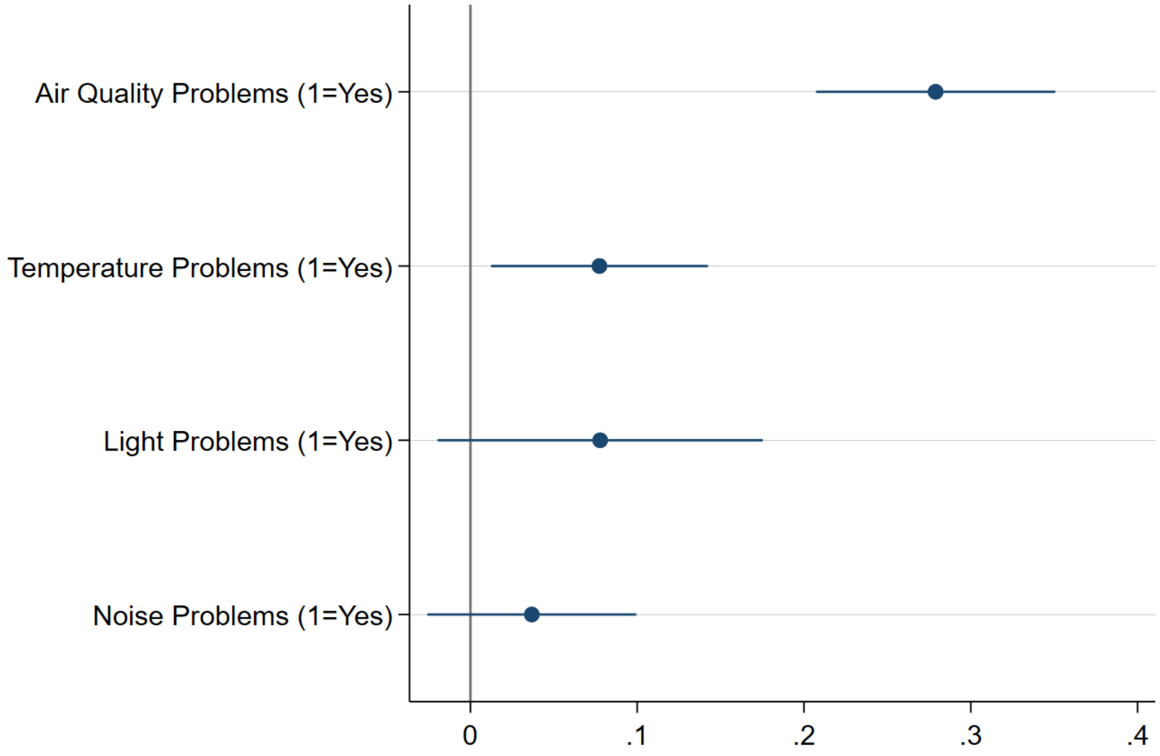

Note: The figure shows the estimated coefficient of the time dummies describing the survey times before (= 0) and after the moving date. The dots represent the point estimates and the bars the 95% confidence intervals. All regressions include the *Elo score* individual fixed effects. The vertical, dashed gray line indicates the moving date. The set of control variables include the average hours worked per week and the layout scales (See Table **??** for the full list).
