# Supplementary material for: Moving to productivity: The benefits of healthy buildings
Source: PLoS One. 2020 Aug 6;15(8):e0236029. doi: 10.1371/journal.pone.0236029 (PMC7410200; doi:10.1371/journal.pone.0236029)
Supplement: Attachment — Submitted filename: response_referee.pdf [file pone.0236029.s004.pdf]

Moving to Productivity: The Benefits of Healthy Buildings

Piet Eichholtz*[*] Nils Kok[†] Juan Palacios[‡]

Response Note to Academic Editor and Referee

In this note, we summarize our revision in response to the main issues raised by the academic editor and the referee. If important ambiguities remain, we stand ready to resolve them.

1 Response to Academic Editor

(i). You used the word "quasi-experiment". I understand people often use natural experiment and quasi experiment interchangeably; I would suggest you use natural experiment. The reason is that relocating to the new building is a decision exogenous to individuals; it is probably a decision by the city government (please clarify if this is true). So for individual workers, relocation is an exogenous shock out of their control, and also the relocation itself is not for the purpose of making workers more healthy, it is probably due to the office space constraint or government's budget situation. A quasi experiment means the setting is an experiment designed for a particular research but for some reasons the design cannot randomly assign subjects to the treatment group. A good reference is chapter 15 "Natural and quasi experiments" in a book "Research methods in practice" 2nd edition by Remler and Van Ryzin, Sage Publications, 2015.

- *We have changed the use of the wording "quasi-experiment" to "natural experiment." In addition, and as described below, we have added a paragraph to better explain the relocation process (it was indeed exogenous and individual employees did not influence their relocation decision).*

(ii). The key of your research setting is to explain clearly how the relocation decision is made. You only vaguely mentioned the selection is based on teams not individuals. More details

[*]Maastricht University, The Netherlands; n.kok@maastrichtuniversity.nl; Corresponding address: Maastricht University, School of Business and Economics, Department of Economics, Tongersestraat 53, 6211 LM Maastricht, The Netherlands. Fax: +31 433 882000.

[†]Maastricht University, The Netherlands

[‡]Massachusetts Institute of Technology, USA; IZA Bonn, Germany

are needed as the second referee is concerned about. For example, if a team is selected, are all members of the team relocated? Are there substantial differences between selected teams and those that did not move? These details do not jeopardize your DID identification strategy as your setting is a natural experiment. This also means you need to describe the background of the new office building and relocation (related to my previous comment) to show it is a natural experiment.

- *Page 5 of the paper now describes in detail the mechanics of the relocation process, which indeed involved functional groups rather than the selection of individuals. We have also cleaned up Table 1 and its corresponding text, to highlight the pre-trends before the move - there are no observable differences in the health and productivity proxies between the treated and non-treated groups.*

(iii). What is the distance between the old and new office building? Does this affect workers' commute behavior?

- *This is an excellent remark. We have added a short description on the new building relative to the existing buildings. In short: the buildings are located quite close to each other, both from the perspective of commute by car and the perspective of commute by public transit. If anything, one of the old buildings is located next to a train station that serves a local commuter train, whereas the new building is located close to an intercity train station - for local commuters the "old" building may have been slightly better for the use of public transit.*

(iv). Describe Figure 1 the survey waves in Section 2.2.

- *Done.*

(v). Typo: first sentence of the 2nd paragraph on p. 9: Figure 3 should be "Figure 2".

- *Done.*

(vi). A recent study shows workplace distraction significantly reduces worker productivity. You may cite it. (Citation: Bialowolski P, McNeely E, VanderWeele TJ, Weziak-Bialowolska D (2020) Ill health and distraction at work: Costs and drivers for productivity loss. PLoS ONE 15(3))

- *Done.*

2 Response to Referee

(i). The health status used in this article are subjective measures, reported by employees. People's subjective observation could be biased due to the building is new and everything looks good, instead of due to improvement in specific dimensions of the environment. Take the temperature quality as an example. The level and volatility of temperature is objectively measurable. The distinction between subjective and objective improvement in the environment is important for policy implications: is it good enough to make the environment "look green" or should the investment be spent on improving the air, temperature, and etc.

- *The referee raises an important and interesting issue: to what extent do we measure perception versus reality. We try to tackle this concern using two different strategies. First, we follow the suggestion of the referee and include objectives measures of environmental conditions in the old and new "green" building. As it turns out, the municipality had some data on the old building, and much more data on the new buildings. While this data is not sufficient for a formal analysis, the results are interesting. Figure A.1 in Appendix A shows that the environmental perception corresponds to actual improvements in objectives measures of air quality and thermal comfort.*

[Insert Figure A.1 About Here]

- *Second, Panel B of Figure 2, a new figure in the paper, displays the changes in satisfaction of employees relocated to the new building. The figure shows clear di erences in changes across environmental dimensions. Individuals in the sample experienced the largest improvement in the perceived air quality; followed by thermal comfort, and experience no significant improvement in their perception of light, privacy, or acoustic comfort.*

(ii). How "green" is the new building? Is there a LEEDS rating for it? Is the effects showed here idiosyncratic to the building studied in this paper or can we extrapolate the effects to other buildings "similarly green"?

- *The paper now includes an extensive description of its "green" features. However, the building is not rated by BREEAM, LEED or another green building scheme. We have included the following paragraph in the paper to explain this issue in more detail: "While the new building was designed to the highest standards of green building principles, it has not applied for a green building certificate such as BREEAM or LEED. In*

the very first stages of the building's design process, the aim was to opt for BREEAM certification. However, at that time, BREEAM did not incorporate building health aspects in its certification process and was solely focused on energy efficiency, and, for example, recycling of building materials. This would have steered the design into an unwanted direction: not enough ventilation (to improve energy efficiency) and recycled, but potentially unhealthy building materials. On top of that, the certification process was deemed expensive, and the municipality thought that money would be better spent on a high-quality interior climate maintenance system, which was indeed installed. The new building has been certified under the "Cradle to Cradle" principle, an attestation to the re-usability of products in the building. In addition, it has won a range of accolades for its sustainable design and performance (3)."

(iii). The authors motivate the study with the tradeoff between the costs associated with improving working environment (say making office building green) and the costs associated with absenteeism (potentially due to sick-building-syndrome). Yet, in the analysis part, these costs and benefits are not explicitly discussed.

- *This is an excellent point. We have included a simple cost-benefit analysis in the conclusion of the paper (and a reference to the outcome of the cost-benefit analysis in the introduction). Part of the cost-benefit analysis had already been constructed by the municipality, and we extended the analysis by including the economic effect of reduced sick leave. This analysis requires some strong assumptions, given that we don't have administrative records, and we have made clear in the paper that it should be interpreted with caution. See below the paragraphs:*

  "Importantly, a mediation analysis shows that the relocation had a significant impact on two proxies for productivity. Job satisfaction increased, and there was a drop in the prevalence of sick leave by 2%. While we don't have access to administrative records, an overly simplified back-of-the-envelop calculation shows that this reduction has meaningful implications for the public finances of the municipality. In 2019, the aggregate salary spent on employees working in the new building was €54 million, for a total of 800 employees. Of those 800 employees, 43% report in sick at least once per year, with an average sick leave of about 5 days per employee per year. The reduction of sick leave by 2% (considering solely the extensive margin, not the changes in length of sick leave), saves the municipality €25,000 per year, which seems small, but at current cost of capital (around 1%) translates into a capitalized value of €2.5 million.

  Of course, the improvement in productivity through reduced sick leave is just a "co

benefit" of the newly constructed building. The city of Venlo did an extensive cost-benefit analysis for the new "green" building. This life-cycle analysis calculated both costs and benefits for a 40-year period. The outcomes of this analysis show a €3.4 million marginal investment in technical installations and healthy materials, in addition to the budget that would have been needed for a conventionally engineered building. The design created savings in building management and exploitation costs, for example maintenance and energy, of approximately €17 million over the 40-year period. Importantly, this cost-benefit analysis only involved proven benefits. Given the uncertainty of any productivity and/or health benefits of the new building (convincing academic evidence for this was lacking at the time), these were not included in the analysis."

(iv). Spillover effects of green building on the neighborhood, on CO2 emission and etc. is a bigger component of the benefits of green building and at the center of the decision about whether private parties should invest in making building green and whether the public/the government should subsidize making building green. The authors need to justify further why they focus on workers subjective feeling of health.

- *There is a large and growing body of literature on buildings and carbon emissions, and the role of buildings (and building owners) in reducing this footprint. We have now included references to this literature in the paper, embedding the paper more firmly in that literature. We also position the health effects of "green" buildings as "co benefits," above and beyond the primary goals of reduction of energy consumption and carbon emissions.*

(v). "Job-related" emotions are not only affected by physical environment but also by social environment. Is it possible that workers take reallocation as a chance to choose new "working neighbor" and thus improve their job-related emotions?

- *Our previous submission was not clear in this regard. We study subjective measures of health, not job-related emotions. We find heterogeneity in the effect on health measures, so if the relocation would generally increase job-related happiness, we would find positive effects across the board.*

(vi). Why not studying the mediation effects of subjective health on absenteeism? For example, regress absenteeism on both the treated*after and the subjective health, to see whether subjective health is the mechanism through which reallocation to a green building influence absenteeism.

- *This is a fantastic suggestion of the referee, which we have embraced wholeheartedly. Using some of the recent literature on mediation analysis (1; 2), we estimated the indirect effect of sick building syndrome (SBS) on sick leave, as well as on job satisfaction. Interestingly (and importantly), the results for the direct effect are zero, but the results for the indirect effect are in line with expectations - reduced sick leave and increased job satisfaction. We included Section 4.4 to describe the analysis and the results, and included Figure 5 to visualize the analysis. We want to reiterate our gratitude to the referee for making this suggestion.*

(vii). While the authors argue that the decision to move is random, the sample who respond to authors' surveys may not be random. It is possible that people who have more emotions to express and or those who have more free time are more likely to respond to the authors survey. This may bias the estimation.

- *To document the strategy of the municipality to relocate workers, we conducted an extensive interview with the local official responsible for the development of the building. Our current version of the manuscript is much cleaner when it comes to describing the process of reallocation of employees, and much clearer when it comes to analyzing pre-trends. See Section 2.1 of the paper.*

References

[1] Hicks, R. and D. Tingley (2011): "Causal mediation analysis," *Stata Journal*, 11, 605–619.

[2] Kosse, F., T. Deckers, P. Pinger, H. Schildberg-Hörisch, and A. Falk (2020): "The formation of prosociality: Causal evidence on the role of social environment," *Journal of Political Economy*, 128, 434–467.

[3] Kraaijvanger (2020): "City hall Venlo. Brief Beyond sustainability. www.kraaijvanger.nl/en/projects/city-hall-venlo/," .

Figure 1: Measurements Environmental Conditions

a. CO_2 Measurements

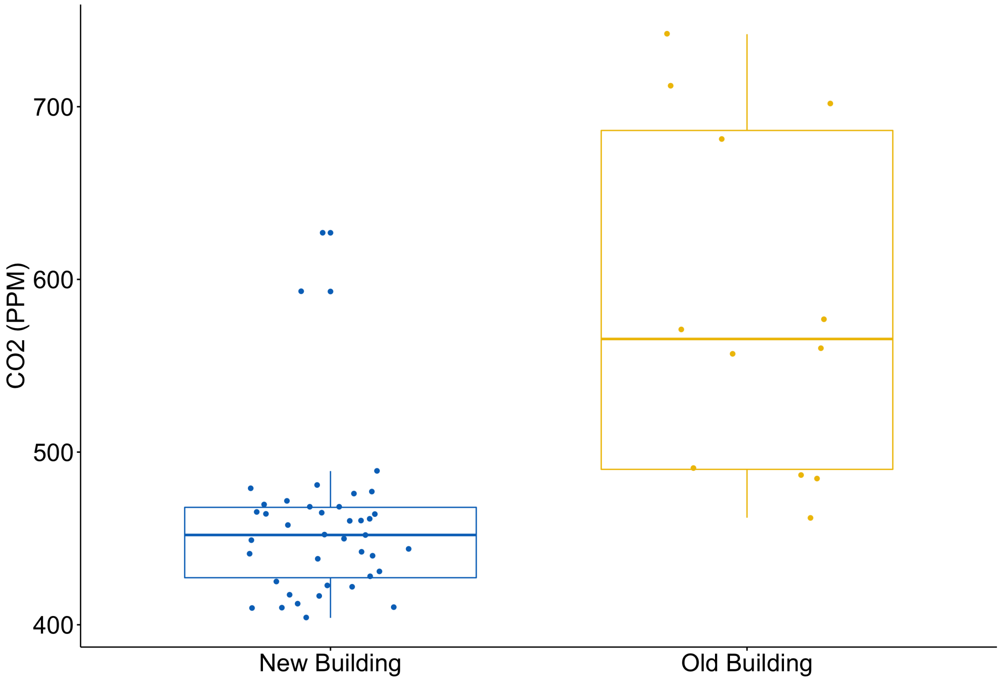

b. Temperature Measurements

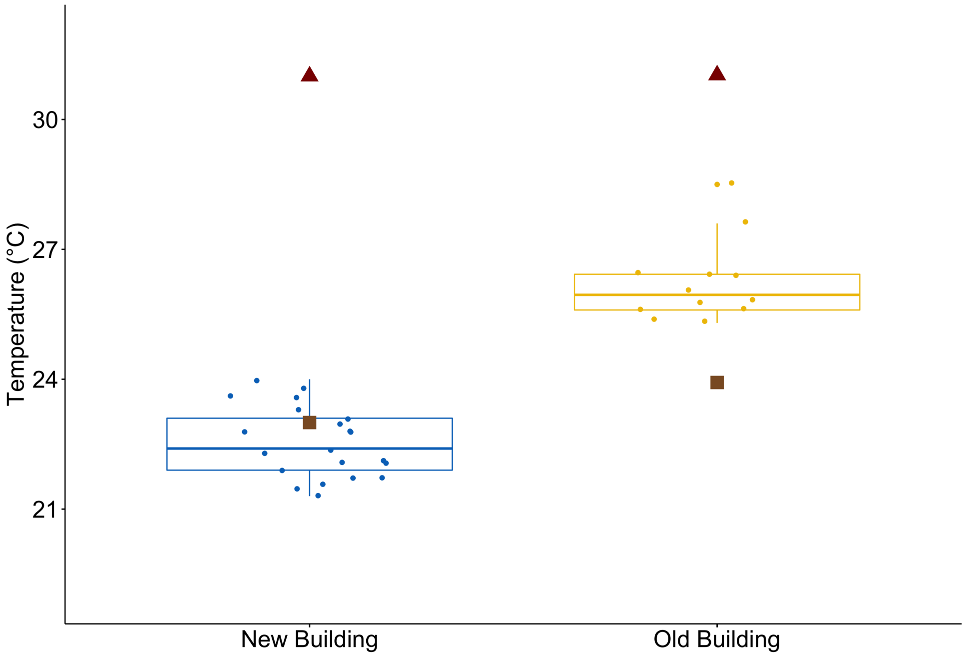

Note: The square (triangle) describes the average (maximum) daily temperature measured by the closest weather station (located in Arcen). Figures based on author's own calculations, based on data retrieved from measurement campaigns undertaken by a professional building surveyor, upon request of the municipality. The measurement campaign in the old building covered four days (before the workers were relocated, from 17/07/2016 to 22/07/2016). In the new building, there is data available from multiple measurement campaigns distributed over all of 2017. For comparison purposes, the distribution of temperatures in the new building is based on a day in 2017 that matches the average peak temperature during the measurement campaign in the old building (21/06/2017). The distribution of CO2 is based on the days that matches the dates in the measurement campaign in the old building to ensure that the occupancy levels are comparable (19/07/2017 to 21/07/2017). Outdoor temperatures are retrieved from The Royal Netherlands Meteorological Institute (KNMI).
